# Aconitine and its derivatives: bioactivities, structure-activity relationships and preliminary molecular mechanisms

**DOI:** 10.3389/fchem.2024.1339364

**Published:** 2024-01-22

**Authors:** Pengyu Zhao, Ye Tian, Yuefei Geng, Chenjuan Zeng, Xiuying Ma, Jie Kang, Lin Lu, Xin Zhang, Bo Tang, Funeng Geng

**Affiliations:** ^1^ School of Clinical Medicine, Chengdu University of Traditional Chinese Medicine, Chengdu, China; ^2^ Guizhou Yunfeng Pharmaceutical Co., Ltd., Qianxinan Buyi and Miao Autonomous Prefecture, China; ^3^ Sichuan Key Laboratory of Medical American Cockroach, Chengdu, China; ^4^ Sichuan Engineering Research Center for Medicinal Animals, Chengdu, China; ^5^ Sichuan Good Doctor Pharmaceutical Group, Chengdu, China; ^6^ Chengdu University of Traditional Chinese Medicine, Chengdu, China

**Keywords:** Aconitine, biological activities, structure-activity relationships, molecular mechanism, diterpenoid alkaloids

## Abstract

Aconitine (AC), which is the primary bioactive diterpene alkaloid derived from *Aconitum L* plants, have attracted considerable interest due to its unique structural feature. Additionally, AC demonstrates a range of biological activities, such as its ability to enhance cardiac function, inhibit tumor growth, reduce inflammation, and provide analgesic effects. However, the structure-activity relationships of AC are remain unclear. A clear understanding of these relationships is indeed critical in developing effective biomedical applications with AC. In line with these challenges, this paper summarized the structural characteristics of AC and relevant functional and bioactive properties and the structure-activity relationships presented in biomedical applications. The primary temporal scope of this review was established as the period spanning from 2010 to 2023. Subsequently, the objective of this review was to provide a comprehensive understanding of the specific action mechanism of AC, while also exploring potential novel applications of AC derivatives in the biomedical field, drawing upon their structural characteristics. In conclusion, this review has provided a comprehensive analysis of the challenges and prospects associated with AC in the elucidation of structure-bioactivity relationships. Furthermore, the importance of exploring modern biotechnology approaches to enhance the potential biomedical applications of AC has been emphasized.

## 1 Introduction

**FIGURE 1:**
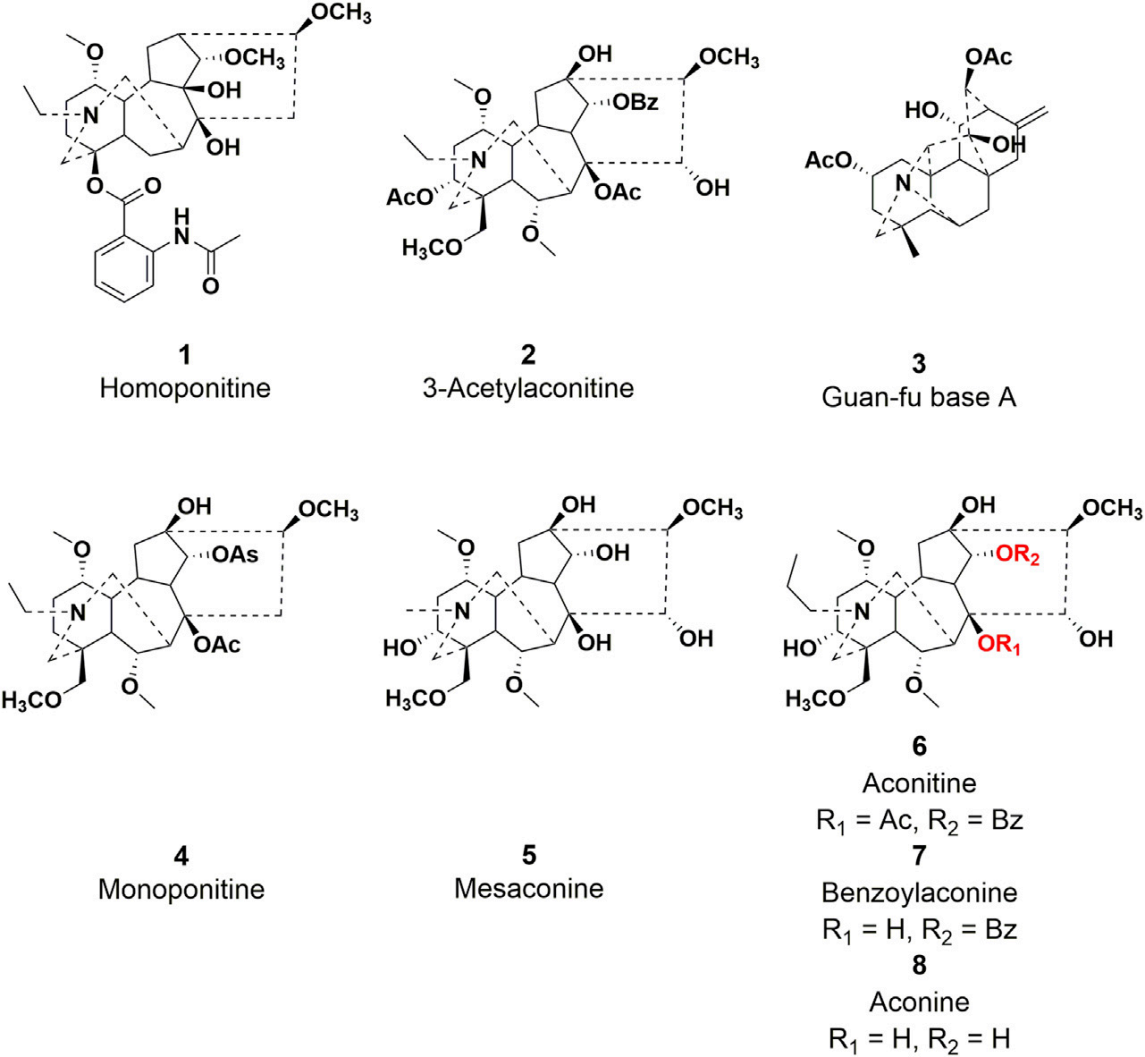
Chemical structures of representative diterpenoid alkaloids **1–8**.

Diterpenoid alkaloids have been identified as the primary active constituents from *Aconitum L* plants among the reported chemical constituents (Khan H et al., 2018). These compounds are a class of heterocyclic compounds resulting from the amination of either tetracyclic or pentacyclic diterpenoids. These compounds can be categorized into C_18_, C_19_, C_20_, and other diterpenoid alkaloids based on their respective skeleton types (Turabekova et al., 2010). To date, four distinct types of diterpenoid alkaloids, namely, homoponitine (**1**), 3-acetylaconitine (**2**), Guan-fu base A (**3**), and bulleyaconitine A (**4**), have been synthesized into pharmaceutical agents and effectively employed in clinical settings (Figure 1). Furthermore, mesaconine (**5**) exhibits a potent cardiotonic and anti-heart failure effect in preclinical studies (Zhou et al., 2021; Tao et al., 2023). Therefore, natural products play a significant role as valuable resources for the development of clinical drugs.

Aconitine (AC, **6**), initially discovered by Manske in 1833 and subsequently structurally elucidated in 1959, serves as a prototypical C_19_-diterpenoid alkaloid and constitutes the principal toxic constituent in more than 250 Aconitum plant species, which have been extensively employed in traditional medicinal practices. Nonetheless, this compound demonstrates pronounced toxicity, primarily exerting its effects on the vagus nerve while concurrently inflicting harm upon the peripheral nerve (Wada et al., 2022). It has been observed that AC possesses an exceedingly low lethal dose (0.2 mg) in humans (Li et al., 2022). Poisoning symptoms primarily manifest in the nervous and circulatory systems, with subsequent occurrence of digestive system symptoms. AC-mediated ventricular tachyarrhythmia and heart arrest are prominent factors contributing to mortality (Li et al., 2022b). Despite the limitations imposed on the clinical development of AC due to its toxicity, researchers have dedicated themselves to addressing this issue through an in-depth investigation of the precise molecular mechanism underlying AC-induced cardiotoxicity. Additionally, they have integrated contemporary pharmaceutical chemistry theory to conduct an analysis of the structure-activity relationship, with the ultimate goal of identifying AC derivatives that exhibit both low toxicity and high efficacy for the treatment of diverse ailments.

**SCHEME 1:**
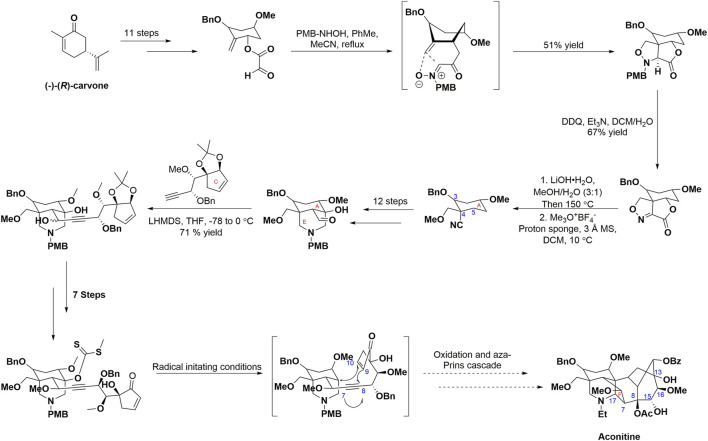
Progress towards the total synthesis of AC. CEs, carboxylesterases.

Structurally, C_19_-diterpenoid alkaloids, to which AC belongs, are characterized by their complex architectural structure, consisting of cage-like compounds that share a hexacyclic ring framework (ABCDEF-ring). These alkaloids typically contain three to nine oxygen substituents (*e.g*., hydroxy, methoxy, and acyloxy groups). Due to their complex structure and significant biological importance, synthetic chemists have shown substantial interest in them during the last 5 decades. Natural medicine chemists (Fernandes et al., 2020; Zhou et al., 2019) reported the most recent endeavor to achieve the total synthesis of AC (Scheme 1). The reported approach successfully synthesized the fully functionallized AE ring system of AC in 27 steps, resulting in an overall yield of 1.64% from (−)-(*R*)-carvone. Specifically, the synthesis was initiated by decorating (−)-(*R*)-carvone to introduce the desired substituents on the A ring. The intramolecular [3 + 2] cycloaddition was facilitated by the *in situ* generation of nitrone from glyoxylate through treatment with *N*-PMB-hydroxylamine, ultimately leading to the formation of a sole diastereomer isoxazolidine. The *N*-PMB group was deprotected and subsequently oxidized using DDQ to yield an imine. The resulting compound underwent hydrolysis, followed by Krapcho decarboxylation, and selective methylation, resulting in the formation of a β-hydroxynitrile. The E ring aldehyde was synthesized through a series of subsequent steps, involving functional group manipulations and an intramolecular Mannich reaction. The incorporation of the alkyne moiety led to the synthesis of propargyl alcohol. Subsequent modifications produced xanthate, which acted as the essential substrate for the pivotal radical cascade reaction, ultimately resulting in the generation of the BD ring systems. The cascade proposed in this study triggers the generation of a radical at C11, which then proceeds to cyclize onto the C10 acceptor and ultimately gets captured by the alkyne moiety. The F ring of AC was successfully synthesized through the implementation of an aza-Prins cascade, representing a significant and conclusive transformation in the proposed route. However, the success rate of compound synthesis in natural product chemistry often encounters challenges (Wangchuk et al., 2018). It is worth noting that solvent extraction is widely employed as the primary method for isolating AC and AC-like terpenoids. Interestingly, researchers within the field of natural product biosynthesis explore the underlying principles that enable Nature to efficiently and selectively synthesize psychoactive compounds (Jamieson et al., 2021). Moreover, the advancement of contemporary molecular biology has facilitated the recognition of AC’s biological properties and health advantages (Tang et al., 2012; Fernandes et al., 2020). In light of its importance, the exploration of structural modification and derivatization of AC to unlock its extensive application potential has emerged as a prominent area of research. Moreover, due to their distinctive structures, aconitine-type C_19_-diterpenoid exhibit a propensity for facilitating the advancement of total synthesis methodologies and structural alterations (Wang et al., 2016). This review will concentrate on the productive accomplishments pertaining to: (i) the biological activities and the underlying bioactive mechanisms of action, (ii) the structural optimization of AC, and (iii) the correlation between bioactivities and the structure-activity relationship of AC derivatives. We are of the opinion that this review will be advantageous in offering constructive recommendations for future investigations on AC.

## 2 The pharmacokinetics of AC

To gain a comprehensive comprehension of the toxicological or pharmacological attributes of AC, it is imperative to initially grasp the *in vivo* pharmacokinetic characteristics of this alkaloid. Typically, AC-infused herbs are orally administered subsequent to decoction, as a means to mitigate any potential adverse effects. Throughout this process, AC experiences two ester hydrolysis reactions, specifically deacetylation at C-8 and debenzoylation at C-14. The C_19_-diterpenoid alkaloid undergoes a conversion process to produce monoestrose-diterpene alkaloid variants, such as phenylmethylaconitine and AC, which are characterized by low toxicity (Gao et al., 2022). Notably, the presence of ester groups in the structure of AC is closely associated with toxicity (He et al., 2019). In contrast to conventional Chinese medicine manual treatment techniques, such as soaking, vulcanization, and boiling, the oral ingestion of AC exhibits a highly intricate biological metabolism, primarily occurring in the liver, stomach, and intestine. Furthermore, the metabolic process involves the catalysis of a hydrolysis reaction by carboxylesterase. Notably, the identification of hydroxylation, deoxidation, and demethylation products through liquid chromatography-mass spectrometry *in vivo* implies the involvement of additional phase I metabolic reactions in this process (Zhang et al., 2016).

**FIGURE 2:**
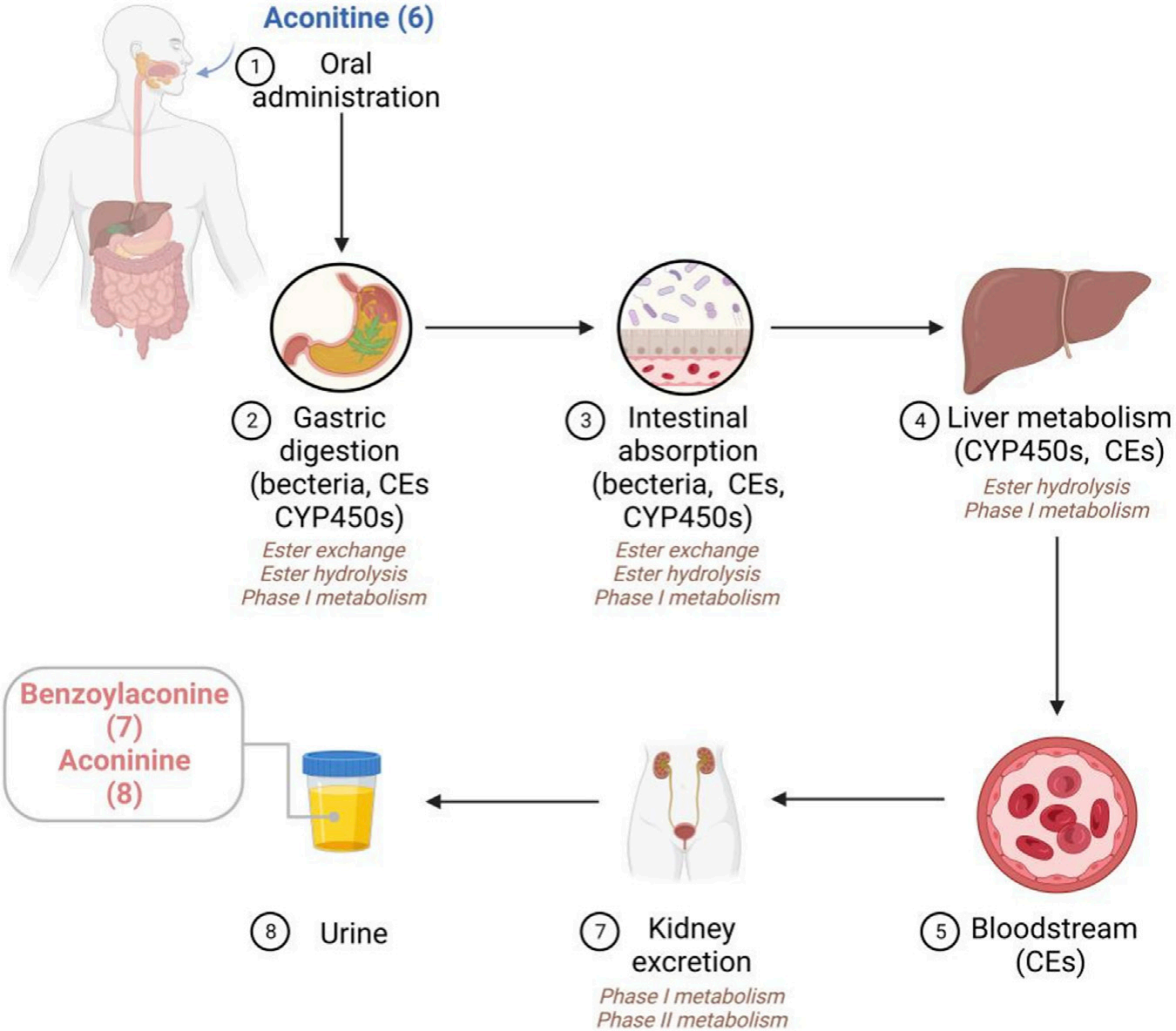
The proposed metabolic pathways of AC *in vivo*.

In this section, an elaborate overview of the metabolic processes of AC in diverse animal species and humans is provided (Chen et al., 2009; Yang et al., 2021b). As shown in Figure 2, the stomach serves as the primary site of metabolism within the digestive system. Specifically, AC primarily undergoes transesterification and phase I metabolic pathways. Previous studies have effectively identified 14 metabolites and 2 ester hydrolysates of AC subsequent to oral administration in *in vivo* models (Zhu et al., 2014). Current evidence indicates that the involvement of CYP2C9 and CYP2C8 enzymes in the gastric region is crucial for the metabolism of AC. While carboxylesterases (CEs) predominates in the liver, intestine, and plasma, it is commonly assumed that CEs facilitates the metabolic pathway of the AC. Specifically, CEs have the ability to hydrolyze a wide range of endogenous substances and xenobiotics that contain ester or amide bonds. Additionally, the majority of identified CEs are categorized into two subfamilies known as CEs1A and CEs2A. Regarding tissue distribution, CEs1A is predominantly present in the human liver with a minor presence of CEs2A, whereas CEs1A is primarily found in the small intestine with minimal expression of CEs1A (Laizure et al., 2013). Unfortunately, despite the existence of studies on the involvement of CEs in AC metabolism, limited reports are available regarding the specific impacts of CEs1A and CEs2A isoforms on AC metabolism. Considering the distinct tissue distribution and substrate-specific affinity of CEs1A and CEs2A, it is postulated that CEs2A may exert a more pivotal role in AC metabolism (Song et al., 2021). However, further comprehensive investigations conducted by researchers are imperative to validate this supposition. Similarly, there remains a hypothesis that CEs in the gastric mucosa promotes the hydrolysis of ester groups within the structure of AC. Moreover, CEs in the intestine may also catalyze ester hydrolysis, as indicated by the structural composition of AC (Zhang et al., 2020b). Furthermore, the intestinal bacterium possesses the capability to secrete a variety of enzymes that play a significant role in the metabolic processes of AC. In the liver, AC primarily undergoes metabolism through phase I metabolic pathways. The phase I metabolism of AC primarily involves the CYP3A4 and CYP3A5 isoenzymes. Additionally, the hydrolysis of AC ester can be facilitated by carboxyesterase and various isoenzymes (CYP3A, CYP1A1, and CYP1A2), emphasizing the complex nature of AC metabolism (Zhu et al., 2013). In the presence of a pH environment conducive to ester hydrolysis, the partially absorbed AC may undergo gradual hydrolysis by CEs to form benzoylaconine (**7**) and aconine (**8**). Notably, a majority of phase I metabolites were observed in the urine. Additionally, minute quantities of phase II metabolites, namely, glucuronic acid and sulfate derivatives, were observed in the urine of *in vivo* models (Li et al., 2016).

Multiple studies have provided evidence that AC and its secondary metabolites follow similar metabolic pathways. Given that CEs are primarily found in the mucosa of the gastrointestinal tract, liver, and plasma, it is probable that the majority of AC and its intermediate derivatives will undergo metabolic conversion into derivatives with benzoyl- or amine-diterpenoid structures. Furthermore, it has been previously demonstrated that the complete elimination of AC is not achieved, and traces of it can still be identified in the blood. As a result, oral administration of AC would result in the circulation of both AC itself and its metabolites. The potential toxicity or effectiveness of administering AC orally may be linked to the three distinct chemical compounds that are produced through a sequence of metabolic processes or alterations.

## 3 Pharmacological activities of AC

### 3.1 Cardiotonic effect

*Aconitum carmichaelii Debeaux* (Fuzi), derived from aconite, occupies a significant role as the second most commonly used herbal medicine in China. Fuzi is consistently incorporated into numerous renowned formulas for both internal and external applications. Nevertheless, there have been documented cases of AC poisoning resulting from the consumption of aconite roots over the past 2 decades. Notably, adverse cardiac events induced by AC represent the most severe clinical manifestations. Hence, there is a growing sense of vigilance regarding the medical application of prescriptions or herbal remedies containing AC. Nevertheless, the ongoing misapplication of herbal medicines containing AC has led to further instances of acute or long-term cardiac toxicity (Wei et al., 2021). According to previous research, a considerable percentage of individuals (around 90%) who suffer from AC poisoning have shown various types of polymorphic ventricular arrhythmias, including ventricular premature beats, ventricular rapid heartbeat and ventricular flutter (Yu et al., 2015). The fatal consequences of AC poisoning often involve these cardiovascular adverse effects. Moreover, in specific autopsy instances of AC poisoning, it has been noted that myocarditis or significant damage to the heart muscle were evident. Additionally, preclinical investigations have demonstrated that AC exhibits cytotoxic properties towards cardiomyocytes, akin to those observed in cases of acute myocardial infarction (Yang et al., 2020). Furthermore, the manifestation of acute AC poisoning often encompasses palpitations, hypotension, bradycardia, and sinus tachycardia, which are characteristic cardiovascular symptoms (Zhao et al., 2016). Regrettably, the absence of a viable antidote for AC-induced cardiotoxic events poses a significant challenge. In contrast, numerous studies have also provided evidence of AC’s possession of cardiotonic properties, which has led to its utilization in the management of cardiovascular disorders (Zhou et al., 2015). Specifically, the injection of extremely small amounts of AC or its derivatives has shown a significant improvement in cardiac function in rats. Consequently, our investigation into the fundamental molecular mechanisms involved in this phenomenon will serve as a valuable contribution towards the advancement of efficacious therapeutic interventions (Liu et al., 2017). The molecular mechanisms through which AC induces cardiotoxicity or cardiotonic efficacy remain ambiguous. Presently, research efforts primarily concentrate on investigating the interplay between AC and ion channels, the cardiac efficacy resulting from AC exposure, and the influence of epigenetic regulation on this phenomenon. In this chapter, we primarily present a comprehensive overview of the cardiotonic mechanism induced by AC, while subsequent chapters will delve into the cardiotoxicity associated with its use.

#### 3.1.1 Molecular mechanisms of AC-mediated cardiotonic effects

**FIGURE 3:**
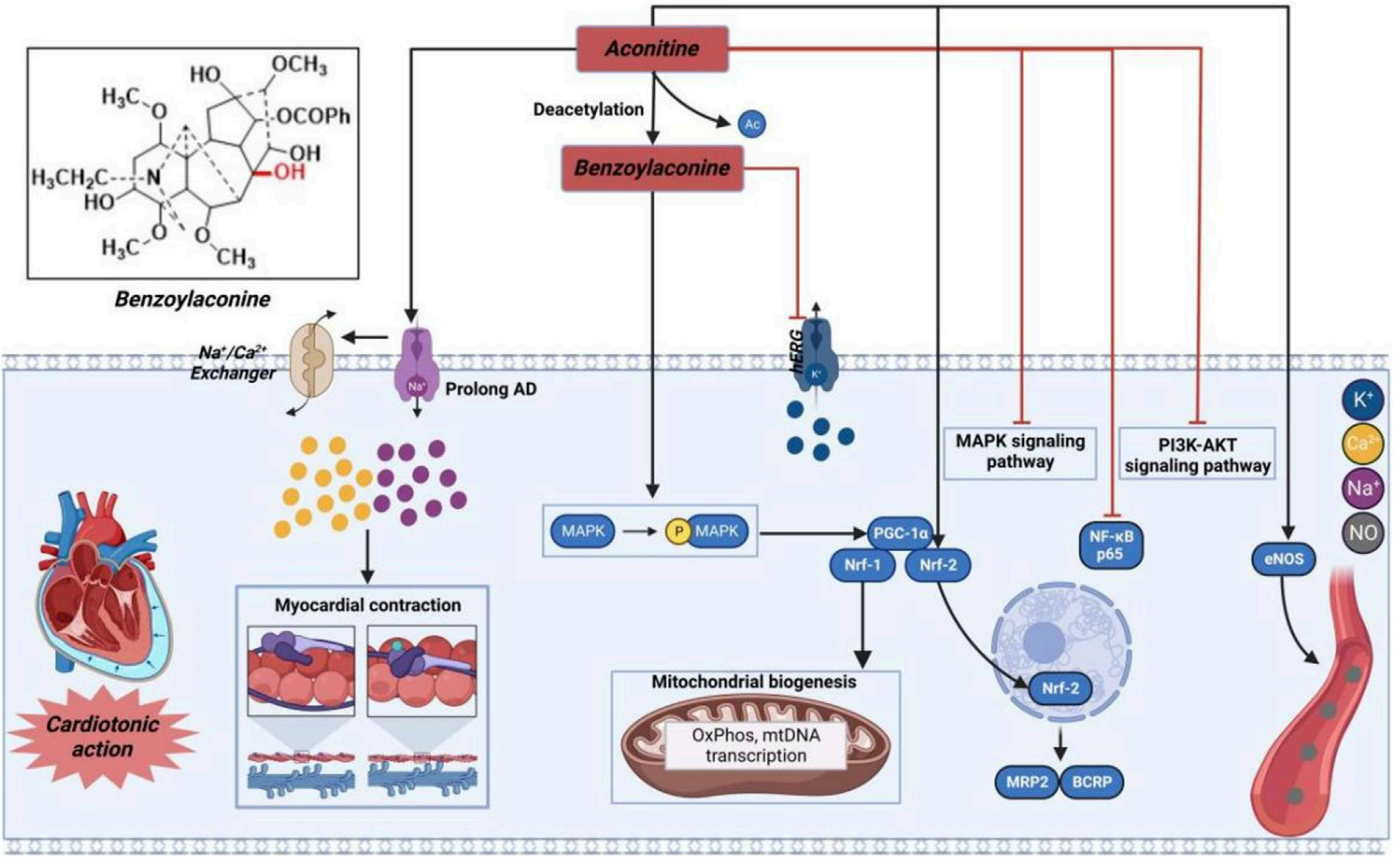
Pharmacological mechanisms of AC-induced cardiac efficacy. Being toxic at high and cardioprotective at low concentrations, AC may possess cardioprotective effect by regulating ion channels and inflammation-related signaling pathway. The black arrow represents the activation of the signalling pathway, and the red horizontal line represents the inhibition of the signalling pathway. AKT, protein kinase B; BCRP, breast cancer resistance protein; eNOS, nitric oxide synthase 3; hERG, human ether-a-go-go related gene; MAPK, mitogen-activated protein kinase; MRP2, multidrug resistance protein 2; Nrf, nuclear respiratory factor; PGC-1α, peroxisome proliferator-activated receptor gamma coactivator 1α; PI3K, phosphatidylinositol-3-hydroxykinase.

As illustrated in Figure 3, AC or its derivatives, like cardiac glycosides, mainly increase cytoplasmic Na^+^-Ca^2+^ concentration to exert a positive inotropic effect (Bi et al., 2020). Simultaneously, scholars have also posited a widely acknowledged perspective suggesting that AC may induce positive inotropic effects through the elongation of the action potential Na^+^ inflow. As previously indicated, the accumulation of Na^+^ can result in the reversal of Na^+^-Ca^2+^ exchange, an elevation in intracellular calcium ions, and an augmentation of cardiomyocyte contractility (Chelko et al., 2019). Moreover, it is worth noting that AC primarily acted as an inotropic agent in pathological instances. Following oral administration, patients with heart failure may experience reduced absorption of AC and its derivatives into the bloodstream, as well as lower affinities for the myocardium cell membrane. As a result, the final amount of these substances in the heart tissue might be considerably reduced, falling within the nanomolar range that is commonly used for therapeutic measurements (Kimura et al., 1994). In addition, AC exhibited cardiotonic effects solely at lower concentrations. The inotropic effectiveness may possibly be negated by the presence of arrhythmia caused by the elevated level of AC (Liu et al., 2012). As a result, many researchers have suggested that, similar to cardiac glycosides, the effects of AC, whether therapeutic or toxic, can differ based on the dosage (Ammar et al., 2013). Hence, the determination of the efficacious dosage of AC can be achieved through the assessment of specific sodium channels or sodium potassium pump (NKA), or alternatively, by evaluating the concentration of sodium or calcium ions within cardiomyocytes. Meanwhile, compound **7**, as the hydrolyzed derivative of AC, exhibits a distinctive property by effectively eliminating the β-acetate on C-8 position, which is widely recognized as the primary contributor to cardiac arrhythmia in AC. In contrast, it acquires an opposing characteristic-antiarrhythmic activity (Nyirimigabo et al., 2015). Moreover, it has been shown that compound **7** efficiently promotes the generation of new mitochondria by exerting a positive influence on the Adenosine 5′-monophosphate (AMP)-activated protein kinase (AMPK) signaling pathway (Yang et al., 2021). Additionally, this molecule has the potential to augment both mitochondrial mass and myocardial contraction in individuals with a failing heart. Gao et al. (2018) further elucidated that the engagement of the AMPK-optic atrophy 1 (OPA1)-ATP5a1 pathway played a role in the mitochondrial turnover and restructuring induced by AC, contributing to a synchronized mitochondrial energy metabolism that facilitated the continuous and organized contraction and relaxation of the heart (Zhang et al., 2019). These findings from both investigations offer alternative insights into the cardiotonic effectiveness observed with the administration of AC or its derivatives.

Prior research has demonstrated that AC can exert a cardioprotective effect in cases of myocardial ischemia through the regulation of nuclear respiratory factor-2 (NRF-2)-mediated anti-inflammatory signaling and nuclear transcription factor-κB (NF-κB)-mediated pro-inflammatory signaling (Liu et al., 2023). Furthermore, AC has the capability to regulate the levels of nitric oxide synthase, thereby enhancing blood circulation in the infarction area and subsequently mitigating myocardial damage resulting from acute myocardial infarction (Mitamura et al., 2002; Wang et al., 2022a). Consequently, AC and its derivatives hold promise as potential pharmacological agents with cardioprotective benefits.

#### 3.1.2 Structure-activity relationship with cardiotonic effects

**FIGURE 4:**
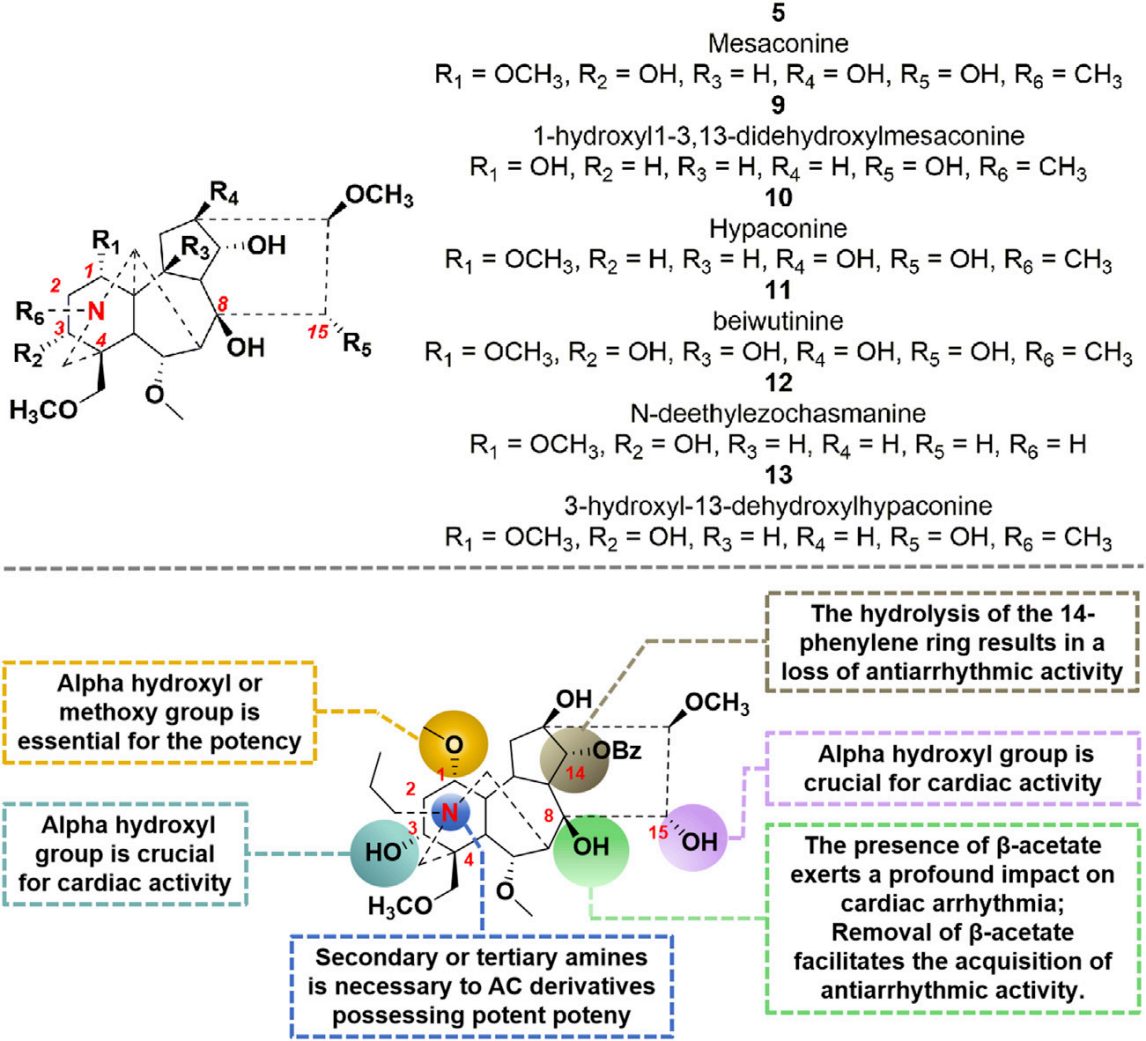
Chemical structures of AC derivatives and structural-activity relationship for the cardiotonic efficacy.

AC has been shown to induce arrhythmia in both preclinical and clinical studies. As mentioned above, the hydrolysis of the β-acetate at C-8 of AC during *in vivo* metabolism results in the formation of compound **7**, which exhibits notable anti-arrhythmia properties. Consequently, the β-acetate at C-8 of AC can be identified as a pivotal cardiotoxic moiety within the molecule. In particular, molecule **7**, with a benzoyl group at position C-14, demonstrates the capacity to selectively hinder the delay rectifier’s K^+^ channel and function as a competitive antagonist against AC-induced arrhythmia (Kiss et al., 2017). However, the hydrolysis decomposition of this compound leads to the decline of its bioactivity, indicating the significant involvement of this aromatic ester substitute in the generation of antiarrhythmic effects by compound **7** (Khan et al., 2018). In 2012, Wang et al. (2022a) conducted a pioneering study on the cardiotropic effect of diterpenoid alkaloids found in aconite. They screened over 60 diterpenoid alkaloids, obtained through synthesis and isolation, for their cardiotropic activity. Furthermore, they investigated the structure-activity relationship of these alkaloids using an *in vitro* frog heart experiment (Wang et al., 2022a). The findings indicate that compounds **5** and **9–13** (Figure 4) exhibit a notable enhancement in cardiac function. Pharmacological investigations have demonstrated that compound **5** exerts a protective influence on isolated rat ischemia-reperfusion heart injury. Moreover, it directly enhances myocardial contractility and improves myocardial diastolic function, while minimally impacting heart rate (Shan et al., 2019). It is noteworthy that GoodDoctor Pharmaceutical Group Co., Ltd. has discovered an efficacious diterpenoid alkaloid monomer, GD-N1702, through scientific investigation on aconite. This compound has been endorsed by the Drug Evaluation Center of China Drug Administration for the treatment of chronic heart failure. Currently, a randomized, double-blind, placebo-controlled Phase I clinical trial is underway to assess the safety, tolerability, pharmacokinetics, and pharmacodynamics of single and multiple doses of this medication in healthy individuals. Collectively, the cardiotonic effects of structure-activity relationship (SAR) of AC derivatives were briefly summarized as follows: (i) The incorporation of specific functional groups at strategic locations within the chemical structure is necessary to AC derivatives possessing potent cardiac activity. As shown in Figure 4, the molecular structures of these active derivatives encompass C (1)-OH (α) or OCH_3_, C (8)-OAc or C (8)-OEt, C (15)-OH (α), secondary amines, or tertiary amines; (ii) The introduction of alpha hydroxyl group at C-3 position could promote the potency; (iii) The lycoctonine type C_19_-diterpenoid alkaloids exhibited no discernible cardiac activity in both *in vitro* and *in vivo* investigations; (iv) The presence of β-acetate at the C-8 position exerts a profound impact on cardiac arrhythmia. Furthermore, removal of β-acetate from the C-8 site facilitates the acquisition of antiarrhythmic activity by the molecule; (v) The hydrolysis of the 14-phenylene ring results in a loss of the molecule’s antiarrhythmic activity.

### 3.2 Antitumor efficacy

#### 3.2.1 Molecular mechanisms of AC-induced antitumor effects

**FIGURE 5:**
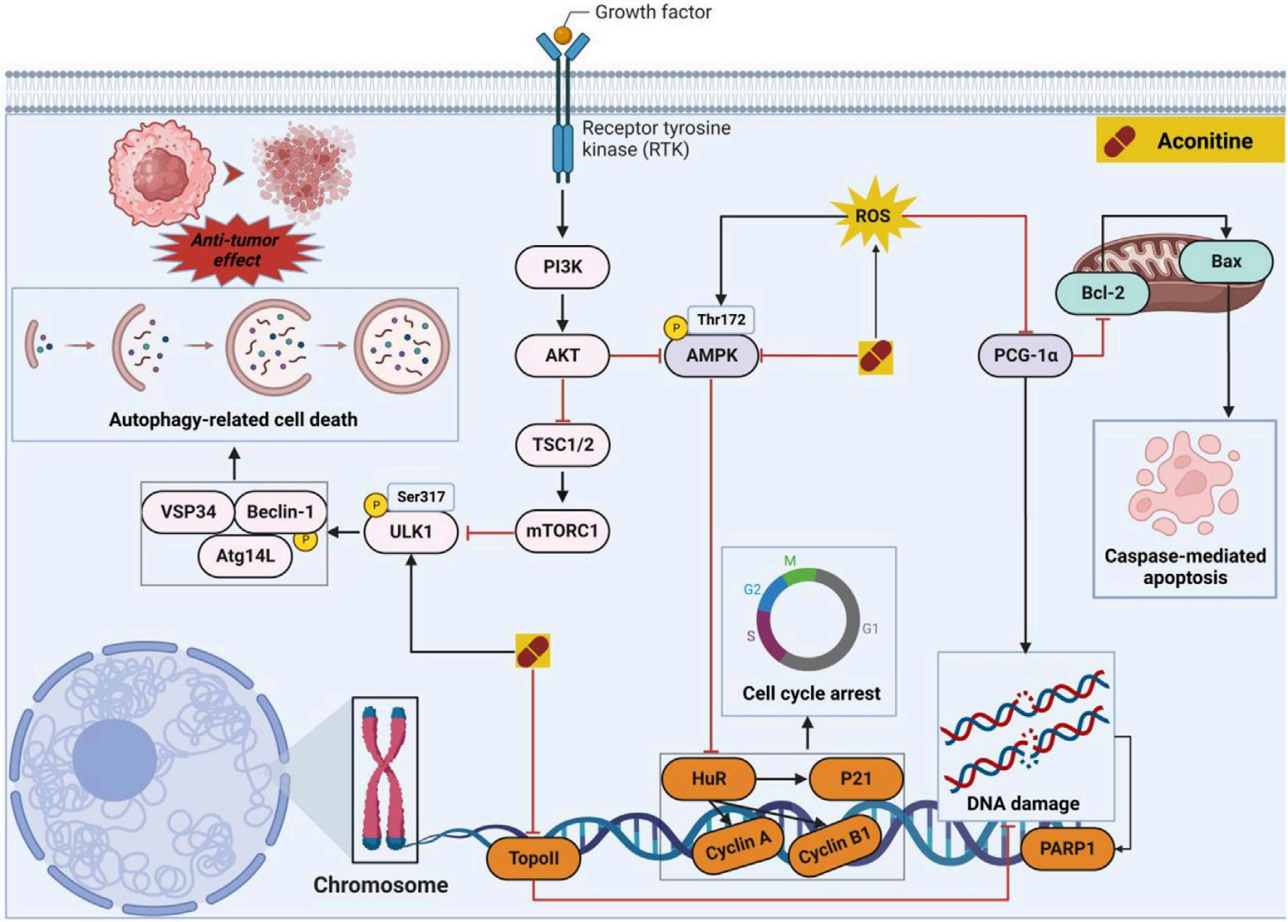
Pharmacological mechanisms of AC-mediated antitumor efficacy. AC has the potential to selectively impact cancer cells through direct modulation of cell cycle- and apoptosis-associated proteins, alongside the regulation of autophagy-mediated cell death. The black arrow represents the activation of the signalling pathway, and the red horizontal line represents the inhibition of the signalling pathway. AKT, protein kinase B; AMPK, 5′-AMP-activated protein kinase subunit gamma-3; Atg14L, Beclin 1-associated autophagy-related key regulator; BAX, apoptosis regulator; Bcl-2, B-cell lymphoma-2; Beclin-1, autophagy-related pseudogene 1; HuR, ELAV-like protein 1; mTORC1, mammalian target of rapamycin complex 1; PGC-1α, peroxisome proliferator-activated receptor gamma coactivator 1α; PI3K, phosphatidylinositol-3-hydroxykinase; PARP1, Poly (ADP-ribose) polymerase 1; ROS, reactive oxygen species; TopoII, topoisomerase II; TSC1/2, serine palmitoyltransferase 2; VSP34, phosphatidylinositol 3-kinase; ULK1, Serine/threonine-protein kinase 1.

For several decades, natural products have served as the primary reservoir of biologically active substances, particularly antitumor lead compounds that exhibit minimal adverse effects (Gao et al., 2018; Yoda et al., 2023). As the 20th century approached its end, AC was recognized for its antitumor properties across various tumor types by targeting multiple pathways (Figure 5). *In vitro* investigations have revealed that AC can impede cell proliferation and facilitate apoptosis-related cell death by negatively modulating the mitogen-activated protein kinase (MAPK)/extracellular regulated protein kinase (ERK) and phosphatidylinositol-3-hydroxykinase (PI3K)/protein kinase B (AKT) signaling pathways (Du et al., 2013). In hepatoma carcinoma MHCC97 cells, the administration of AC demonstrated a significant inhibitory effect on cell proliferation and invasion by modulating the phosphorylation of MAPK. Additionally, AC facilitated the accumulation of reactive oxygen species (ROS), leading to oxidative DNA damage. Consequently, the presence of ROS further stimulated the phosphorylation of AMPK. Previous studies have indicated that the activation of AMPK is closely linked to cell cycle arrest, which is mediated by the activation of p53 and subsequent induction of p21 (Ge et al., 2022). Interestingly, AC has the ability to stimulate the upregulation of pro-apoptotic protein (*e.g.*, Bax) through the accumulation of reactive oxygen species (ROS), subsequently initiating the apoptotic pathway in tumor cells (Xiang et al., 2023). Topoisomerase IIα (Top IIα) is a crucial nuclear enzyme involved in various DNA processes such as replication, transcription, recombination, and chromosome condensation. Among the various categories of antitumor medications, Top IIα poisons are renowned for their exceptional effectiveness. The researchers conducted a study that showcased the remarkable antitumor efficacy of AC against both MCF-7 and MCF-7/ADR cells, with IC_50_ values of 7.58 and 7.02 μM, respectively. By means of molecular docking and topo inhibition tests, it was ascertained that AC functions as a discerning inhibitor of Top IIα. Consequently, AC hinders DNA repair processes and triggers DNA damage, ultimately resulting in the demise of tumor cells (Luan et al., 2022). Finally, autophagy has been widely acknowledged to play a significant role in diverse tumor progression. The administration of AC exhibited a substantial elevation in AMPK^Thr172^ and ULK1^Ser317^ phosphorylation, thereby substantiating the induction of autophagy-associated cell death through the activation of AMPK and subsequent ULK1 phosphorylation (Wang et al., 2022b). Prostate cancer cells have been observed to potentially evade androgen deprivation therapies through the amplification of intracrine androgen synthesis within the prostate (Batra et al., 2020). Presently, the main focus of prostate cancer treatments lies in targeting androgen synthesis and/or androgen receptor (AR) signaling (Mellado et al., 2022). A study conducted revealed that the administration of 50 μM AC for a duration of 24 h effectively hindered androgen synthesis by suppressing the expression of crucial enzymes, such as Scarb1, Hsd3b1, Cyp17a1, and Hsd17b3 (Wang et al., 2019). Collectively, AC demonstrates potential as an antitumor agent. However, it is imperative to acknowledge the cardiotoxicity associated with varying concentrations of this compound.

#### 3.2.2 Structure-activity relationship with antitumor efficacy

**FIGURE 6:**
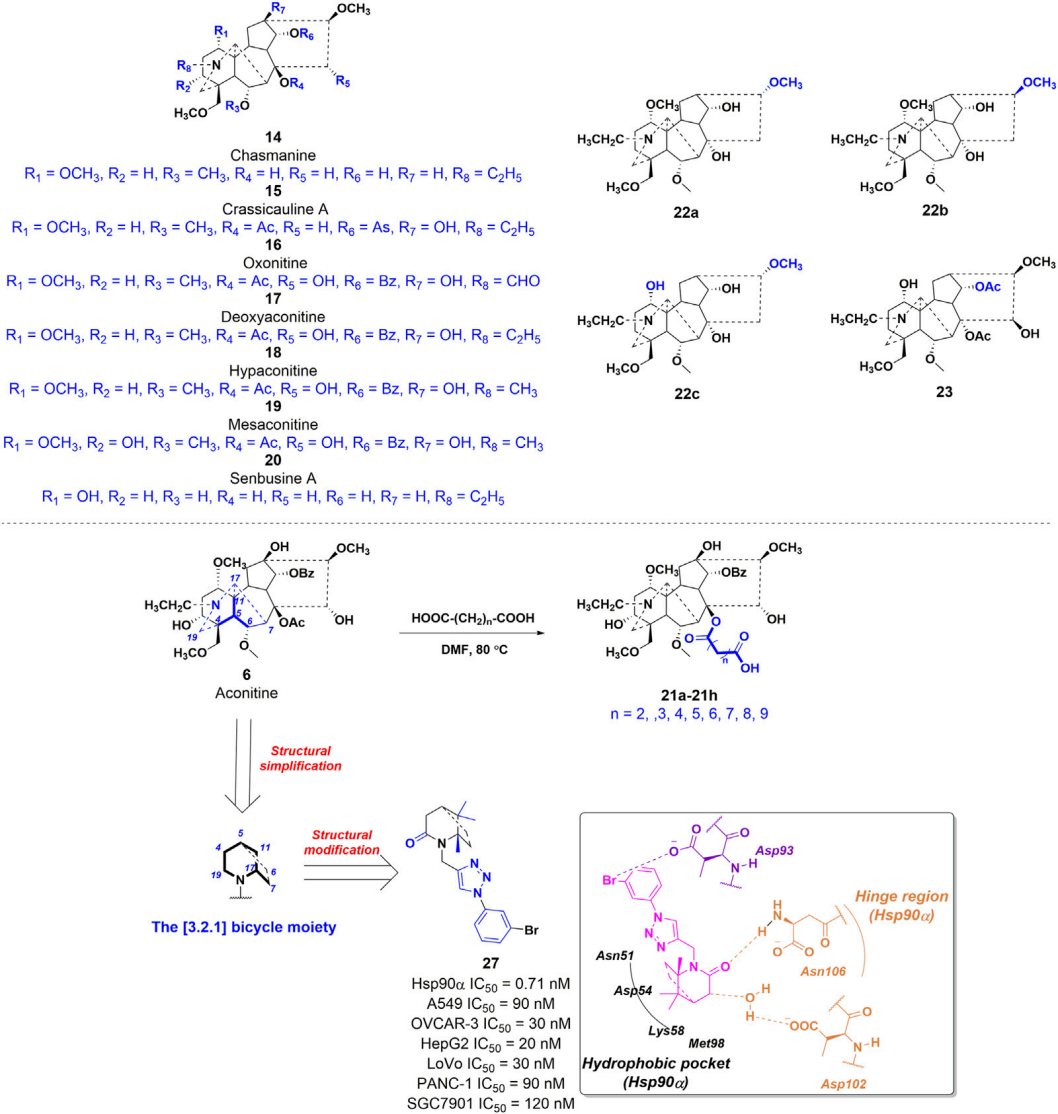
Chemical structures of AC derivatives **14–20**, **21a-h**, **22a-c**, **23**, and **27** with antitumor activities.

**SCHEME 2:**
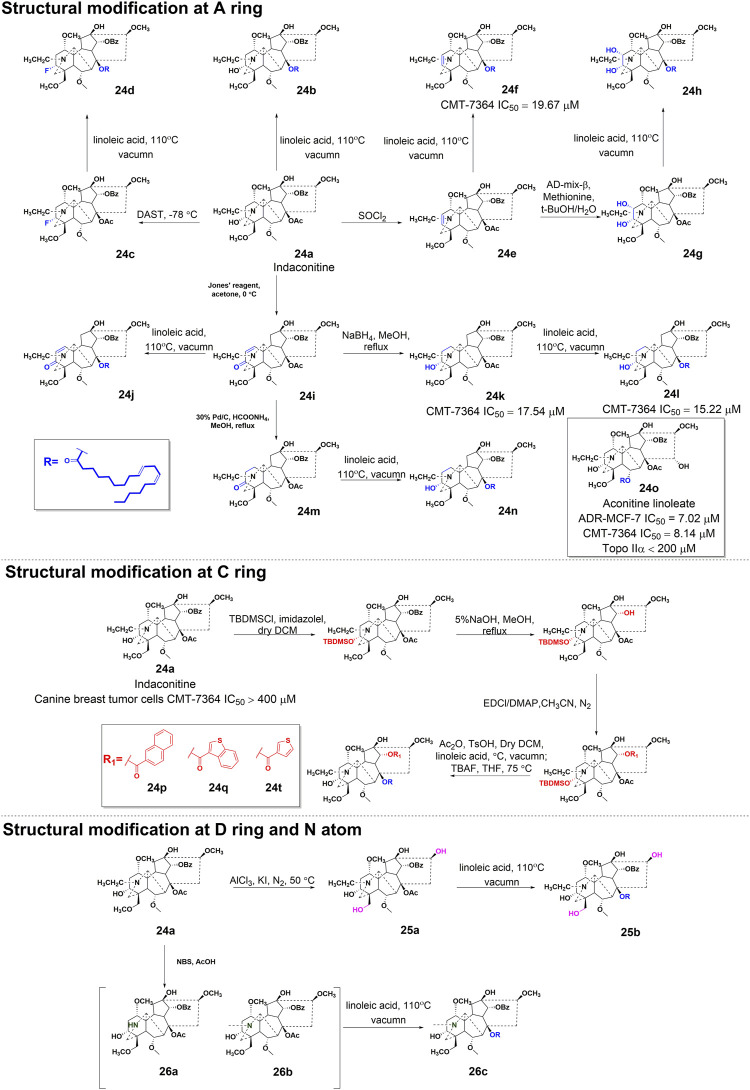
The synthetic route of AC derivatives **24a-q**, **25a-b**, and **26a-c**.

**FIGURE 7:**
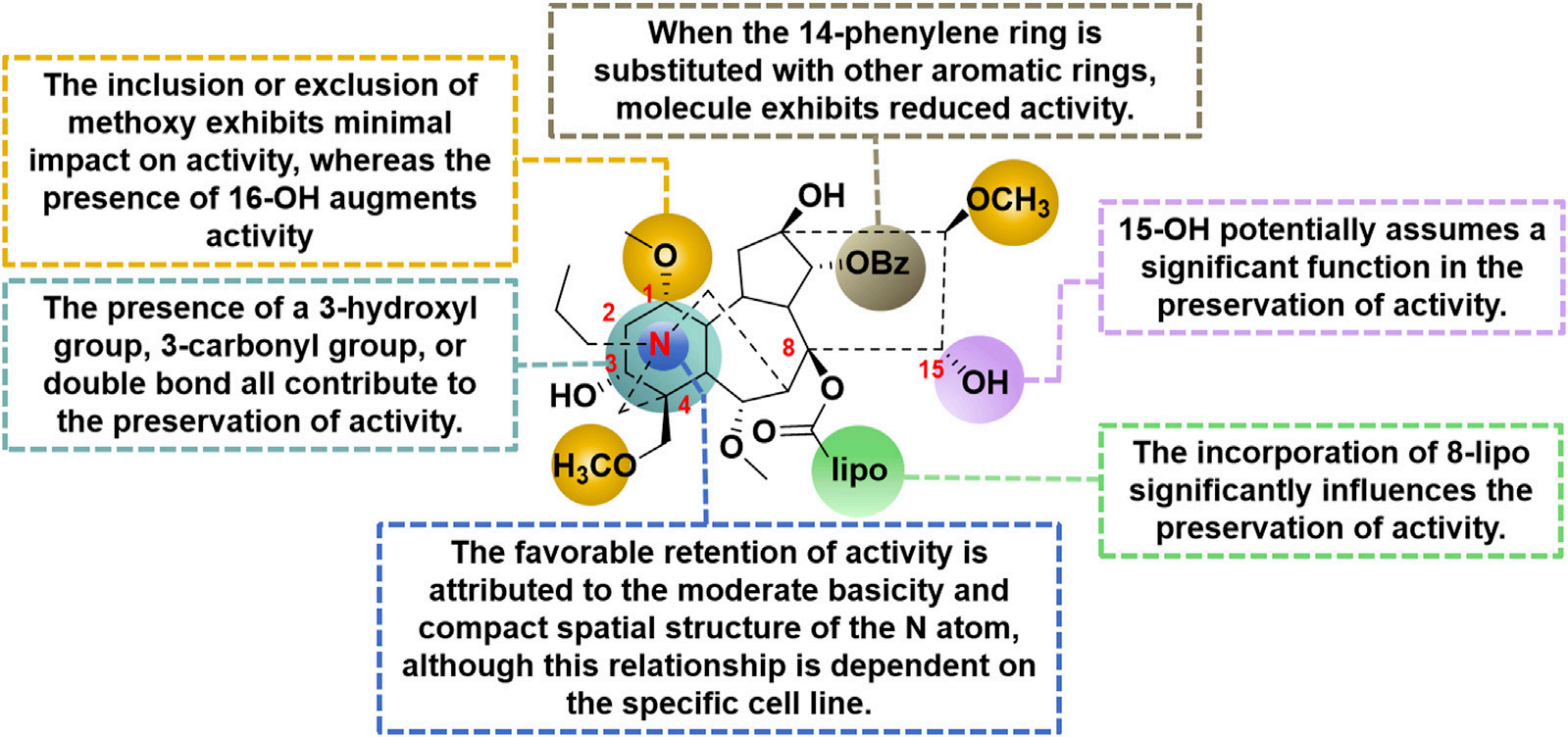
The structural-activity relationship of AC derivatives for the antitumor efficacy.

In a study conducted in 2012, eight C_19_-diterpenoid alkaloids (AC and **14–20**) were evaluated for their efficacy against various tumor cell types (Hu et al., 2021). Notably, AC, **16**, and **17** (Figure 6) exhibited the highest level of potency against HCT8, MCF-7, and HepG2 cell lines, respectively, which are associated with human colon adenocarcinoma, human breast cancer, and human hepatoblastoma. Furthermore, a previous study examined the potential of AC to reverse multidrug resistance in drug-resistant human oral squamous cell carcinoma (KBv200). The findings revealed that AC exhibited a modest inhibitory effect on KBv200 cell lines, with an IC_50_ value of 224.91 μg/mL. This suggests that AC may serve as a reversing agent for multidrug resistance, enhancing the efficacy of vincristine in eliminating tumor cells. Nevertheless, the precise mechanism through which AC reverses resistance remains poorly elucidated (Wada and Yamashita, 2019). In the same year, a series of mono-[*O*-(14-benzoylaconine-8-yl)]esters **21a–h** (Figure 6) were discovered by incorporating alkyl linker chains of varying lengths into the 8-*O*-azeloyl-14-benzoylaconine scaffold (Chodoeva et al., 2012). Furthermore, the antitumor activities of these compounds were evaluated against A549, MCF-7, and HCT-15 cell lines using the MTT method. Regrettably, these compounds exhibited no significant *in vitro* anti-proliferative activity, with IC_50_ values exceeding 60 μM. In 2014, compounds **22a–c** (Figure 6) were isolated from *Aconitum taipeicum*. Notably, compound **22a** exhibited significantly stronger inhibitory effects on HL-60 and K562 cell lines compared to adriamycin (Xu et al., 2010). Additionally, a separate investigation revealed that compound **22a** effectively impeded the proliferation of HepG2 cell lines, with its efficacy being dependent on both dosage and duration of exposure. Intriguingly, at higher concentrations, compound **22a** induced apoptosis-associated cell death in HepG2 cells (Zhang et al., 2014). In 2017, medicinal chemists identified compound **23** (Figure 6), which exhibited a moderate inhibitory activity against SK-OV-3 cell lines, with an IC_50_ value of 43.78 μM (Ren et al., 2017). AC linoleate (**24o**), a lipo-diterpenoid alkaloid derived from *Aconitum sinchiangense* W. T. Wang, was discovered to possess moderate antiproliferative properties against MCF-7 and MCF-7/ADR cells. The IC_50_ values for **24o** were determined to be 7.58 μM and 7.02 μM for MCF-7 and MCF-7/ADR cells, respectively. Additionally, *in vitro* assays revealed that **24o** induced cytotoxicity in MCF-7/ADR cells by arresting the cell cycle at the G0/G1 phase (Jin et al., 2023). As shown in Scheme 2, the aforementioned research group successfully identified a range of structurally diverse derivatives of AC by implementing modifications on the A-ring, C-ring, D-ring, and N-atom of the AC skeleton (Zhang et al., 2023). Specifically, The 3-OH group in the A-ring structure underwent fluorination, elimination, oxidation, reduction reactions, and the addition of a 2-OH group, resulting in the formation of compounds **24c**, **24e**, **24g**, **24i**, **24k**, and **24m**. Subsequently, these compounds underwent an ester exchange reaction, leading to the formation of compounds **24b**, **24d**, **24f**, **24h**, **24j**, **24l**, and **24n**. Additionally, compounds **24p**, **24q**, and **24t** were synthesized through a series of sequential steps, involving the protection of the 3-OH group, hydrolysis of the 14-benzoyl moiety, introduction of the naphthalene ring, benzothiophene ring, and thiophene ring at the C14 position, followed by deprotection and ester exchange. Then, compounds **25a** and **25b** were synthesized through the elimination of the methyl group at positions C16 and C18, followed by an ester exchange reaction. Finally, compound **26a** was obtained by eliminating the nitrogen ethyl group from indaconitine (**24a**), while compound **26c** was formed through the ester exchange of mesaconitine **26b**. Comparative analysis revealed that compounds **24f**, **24k**, **24l**, and **24o** exhibited superior inhibitory potency against canine breast cancer cells CMT-7364, as compared to the prototype compound **24a** (IC_50_ > 400 μM). The IC_50_ values for compounds **24f**, **24k**, **24l**, and **24o** were determined to be 19.67, 17.54, 15.22, and 8.14 μM, respectively. Moreover, **24o** could arrest the cell cycle of CMT-7364 cells in the G0/G1 phase, and exert the anti-proliferative efficacy through inducing apoptosis. Furthermore, this research group provided a comprehensive summary of the structure-activity relationship (SAR) pertaining to the anti-tumor proliferation activity of AC derivatives. Specifically, cellular effectiveness of SAR were briefly summarized as follows. (i) The moderate basicity and compact spatial structure exhibited by the N atom contribute to the retention of activity; (ii) The introduction of hydroxyl, carbonyl, or alkene at the 3-position enhances the biological activity; (iii) The incorporation of 8-lipo demonstrates a significant impact on the preservation of activity; (iv) The involvement of 15-OH potentially holds a crucial position in the preservation of activity; (v) The substitution of other aromatic rings on the 14-phenylene ring leads to a decrease in activity; (vi) The impact of the presence or absence of methoxy at the 16-position on activity is minimal, whereas the introduction of hydroxyl has the potential to augment activity (Figure 7).

In the field of drug development, it is widely recognized that natural products often possess intricate structural frameworks, pharmacokinetic profiles that are not conducive to favorable outcomes, and a lack of drug-like properties (Bekhit et al., 2018). Consequently, a valid and meaningful approach in this process involves the simplification of complex natural product structures while preserving the desired biological activity (Alizadeh and Ebrahimzadeh, 2022). This strategy has proven successful in the optimization of natural product leads, resulting in the development of numerous marketed drugs and drug candidates, such as analgesics derived from morphine (Kumar and Jaitak, 2019). In 2021, compound **27** was identified as a potent inhibitor of heat shock protein α (Hsp90α) (Zhang et al., 2021). The compound demonstrated an IC_50_ value of 0.71 nM, indicating its high inhibitory activity. This compound was developed by simplifying and modifying the AC scaffold, as shown in Figure 6. Specifically, the investigations into the correlation between molecular structure and toxicity have indicated that the presence of hydrophilic substituents at positions C2 and C3 in the A ring, as well as positions C8 and C15 in the C ring, may potentially contribute to neurological toxicities (Wang et al., 2018). According to this study, their design strategy is centered on the preservation of the simplified E-F ring framework as a 2-azabicyclo [3.2.1] octane, with particular emphasis on maintaining the integrity of the C1 and C10 methyl groups, which are highly conserved. Additionally, they underscore the pivotal role played by the carbonyl group at C19 in biological function within their design. Subsequently, computer-aided drug design techniques based on molecular structure were employed, wherein the compound was modified using a fragment growing approach to incorporate a lipophilic phenyl group and connect it to the N2 atom of the 2-azabicyclo [3.2.1] octane core through a 1,2,3-triazole linkage. *In vitro* studies have demonstrated that **27** exhibits significant antiproliferative effects against various tumor cells, with its potency observed in the low nanomolar range. Molecular docking studies revealed that 2**7** effectively occupied the deep ATP-binding pocket of Hsp90α. Notably, the lactam group of **27** formed a hydrogen bond with Asn106, while the azabicyclo group was connected to Asp102 through a water bridge. Moreover, the central pharmacophore [(1*S*,5*S*)-1,8,8-trimethyl-2-azabicyclo (3.2.1)octan-3-one] has the potential to establish hydrophobic interactions with multiple residues, such as Met 98, Lys58, and Asp54. The results of *in vitro* assays revealed that **27** exhibited the ability to induce cell apoptosis and cause a prolonged G1/S-phase in tumor cells. Furthermore, in the xenograft model conducted *in vivo*, compound **27** demonstrated significant efficacy in inhibiting the growth and size of colon tumors. In general, the process of simplifying the structure of natural products is a crucial approach in the identification of active conformations, leading to the discovery of novel molecular entities.

### 3.3 Antiinflammatory efficacy

#### 3.3.1 Molecular mechanisms of AC-induced antiinflammatory effects

**FIGURE 8:**
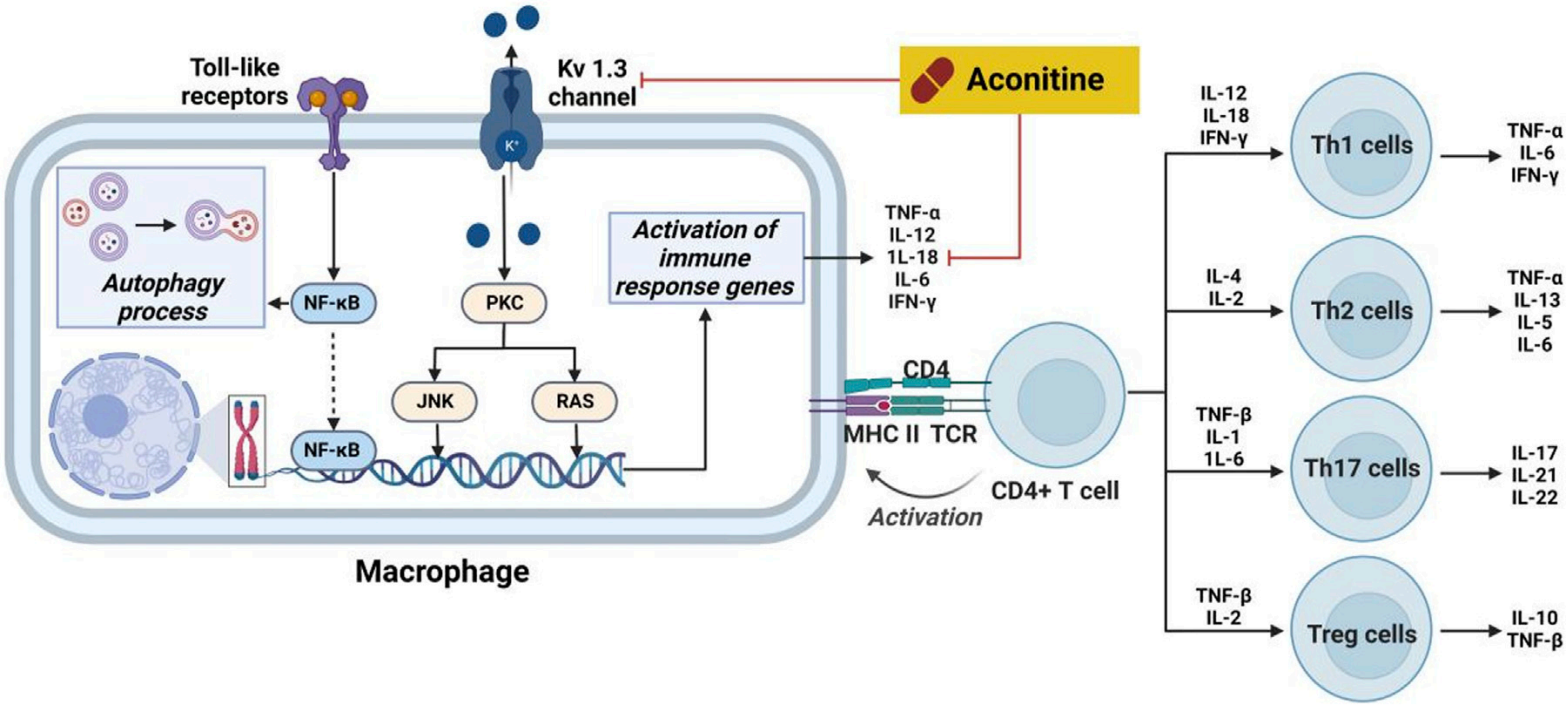
Pharmacological mechanisms of AC-mediated antiinflammatory efficacy. AC possesses anti-inflammatory activity through regulation the expression of cytokines. The black arrow represents the activation of the signalling pathway, and the red horizontal line represents the inhibition of the signalling pathway. IFN-γ, interferon γ; IL, interleukin; JNK, stress-activated protein kinase; MHC II, HLA class II histocompatibility antigen; NF-κB, nuclear transcription factor-κB; PKC, protein kinase C; TCR, pre T-cell antigen receptor; TNF-α, tumor necrosis factor-α.

Inflammation is a complex physiological reaction of bodily tissues to harmful stimuli, including pathogens, injured cells, and irritants (Wu et al., 2021). The primary objective of the inflammatory response is to eliminate exogenous stimuli, eliminate impaired cells and tissues, and initiate the process of autoregeneration (Lichota et al., 2019). In essence, inflammation represents an innate defense mechanism. However, it should be noted that inflammation can also trigger allergic reactions within the body’s immune system, leading to the formation of inflammatory lesions. Furthermore, Previous studies have demonstrated that an overabundance of pro-inflammatory cytokines can induce inflammatory process, consequently modifying the immune response within physiological condition. Notably, cytokines such as interleukin-1β (IL-1β), interferon γ (IFN-γ), IL-5, IL-6, IL-13, IL-17 and tumor necrosis factor-α (TNF-α), which are produced by inflammatory cells and resident macrophages, play a significant role in the onset and progression of inflammatory diseases (Salehi et al., 2019). The inhibitory effects of aconitine-type C_19_-diterpenoid alkaloids on inflammation were elucidated by targeting specific cytokines and pathways (Figure 8).

Prior research has documented the therapeutic properties and underlying mechanisms of AC in the context of systemic lupus erythematosus. Specifically, following a 2-month treatment of AC in mice induced with pristine, notable enhancements were observed in both the overall wellbeing and histopathological state of the kidneys (Li et al., 2017). Concurrently, there was a significant reduction in glomerular leukocyte count, serum anti-DSDNA antibody levels, and IgG deposition. Furthermore, a decrease in levels of IL-17a, IL-6, and PGE2 was observed in AC-stimulated mice. Furthermore, mice subjected to AC treatment exhibited diminished levels of these cytokines. This inhibition subsequently leads to a reduction in kidney inflammation and the suppression of B cell activation. Nevertheless, AC exerted a time- and concentration-dependent inhibitory effect on the amplitude of delayed-rectifier K^+^ current (Wu et al., 2011). Furthermore, AC demonstrated a time- and concentration-dependent inhibition on the amplitude of delayed-rectifier K^+^ current. Furthermore, AC exerted inhibitory effects on the activity of Kv 1.3 channels, thereby impacting the immune regulation associated with these channels in the immune system (Xu et al., 2019). Furthermore, AC demonstrates the ability to impede the growth of rheumatoid arthritis fibroblast-like synoviocytes (RA-FLS) and prompt apoptosis in a concentration-dependent manner, potentially linked to autophagy levels during excessive activation of RA-FLS (Jiang et al., 2022). The therapeutic efficacy of AC in immune-related disorders is ascribed to its capacity to modulate IL-6 and TNF-α levels, alongside its impact on NF-κB signaling pathways (Zeng et al., 2016).

#### 3.3.2 Structure-activity relationship with antiinflammatory efficacy

**FIGURE 9:**
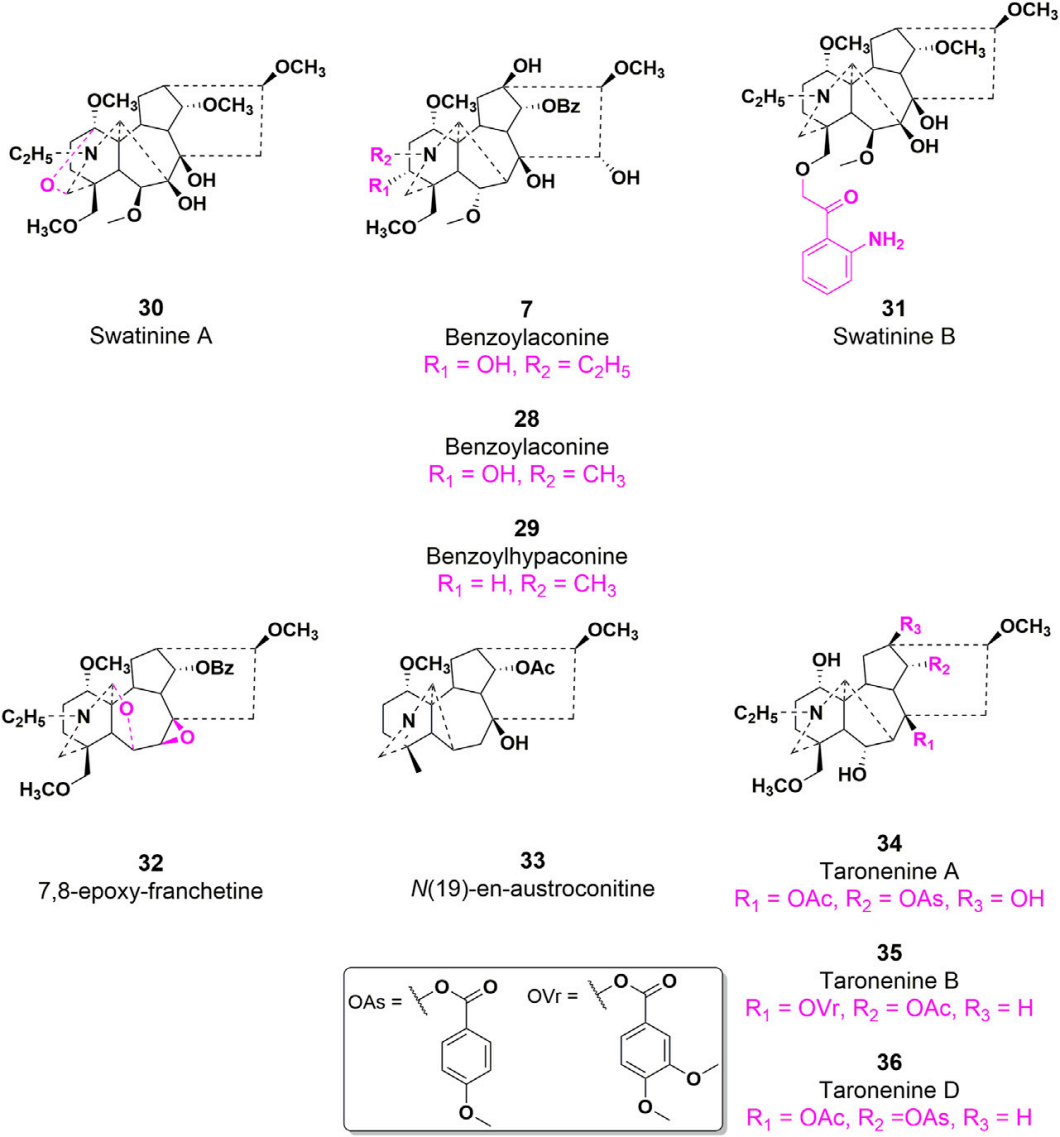
Chemical structures of AC derivatives **28–36** with antiinflammatory efficacy.

*Aconitum carmichaelii Debeaux*, a traditional Chinese medicine, is employed for the management of rheumatoid arthritis due to the presence of diterpenoid alkaloids, which exhibit notable anti-inflammatory and antipyretic properties (Fu et al., 2022). On the other hand, AC has the ability to suppress toe swelling induced by carragetin, histamine, and 5-HT in rats, as well as ear swelling caused by xylene in mice. Additionally, it can enhance the depth of diverse bodily fluids resulting from augmented capillary permeability triggered by histamine and 5-HT (Shang et al., 2010). As shown in Figure 9, the effects of compounds **7**, **28**, and **29** on the *in vitro* proliferation of mouse macrophage RAW264.7 cells were investigated (Li et al., 2023). The results indicated that all three compounds demonstrated the capacity to inhibit the expression of cytokines, such as TNF-α and IL-6, in RAW264.7 cells that were induced by lipopolysaccharide (LPS). In 2014, compounds **30** and **31** exhibited notable anti-inflammatory activity in the nitrotetrazolium chloride detection model involving activated neutrophils, as evidenced by their respective IC_50_ values of 25.82 and 38.71 μg/mL. These values were comparable to the anti-inflammatory potency of indomethacin (IC_50_ = 42.02 μg/mL), a widely recognized positive control (He et al., 2018). Moreover, Compounds **31** and **32** exhibited limited inhibitory effects (27.3% and 29.2%, respectively) on nitric oxide production in RAW264.7 macrophages stimulated by LPS. Conversely, compounds **33**, **34**, and **35** demonstrated moderate inhibitory effects on IL-6 production in RAW264.7 macrophages induced by lipopolysaccharide, with IC_50_ values of 29.60, 18.87, and 25.39 μg/mL, respectively. In comparison, the positive control, dexamethasone, exhibited an IC_50_ value of 15.36 μg/mL (Li et al., 2019). Despite the extensive elucidation of the anti-inflammatory properties of AC, there is a scarcity of reports on the corresponding characteristics of AC derivatives. Consequently, the elucidation of the SAR pertaining to the anti-inflammatory efficacy of AC continues to pose challenges. In general, further inquiries are imperative to explore more effective AC-based drugs and elucidate the fundamental molecular mechanisms underlying their therapeutic potential in the management of inflammation-associated ailments.

### 3.4 Analgesic efficacy

#### 3.4.1 Molecular mechanisms of AC-induced analgesic effects

Pain is an exceedingly disagreeable sensation and emotional experience that not only induces physical discomfort but also profoundly impacts individuals’ mental and psychological wellbeing, particularly when it reaches a severe level (Turnaturi et al., 2019; Zhu et al., 2021). Consequently, the alleviation and management of pain have perpetually remained a paramount concern within the realm of human existence. The endocannabinoid system exhibits potential as a viable avenue for alleviating pain due to the fact that endocannabinoid toxins serve as intrinsic ligands for the pain-associated receptors, namely, cannabinoid receptors 1 and 2 (CB1 and CB2), as well as the transient receptor potential cation channel subfamily V member 1 (TRPV1) (Hu et al., 2023).

**FIGURE 10:**
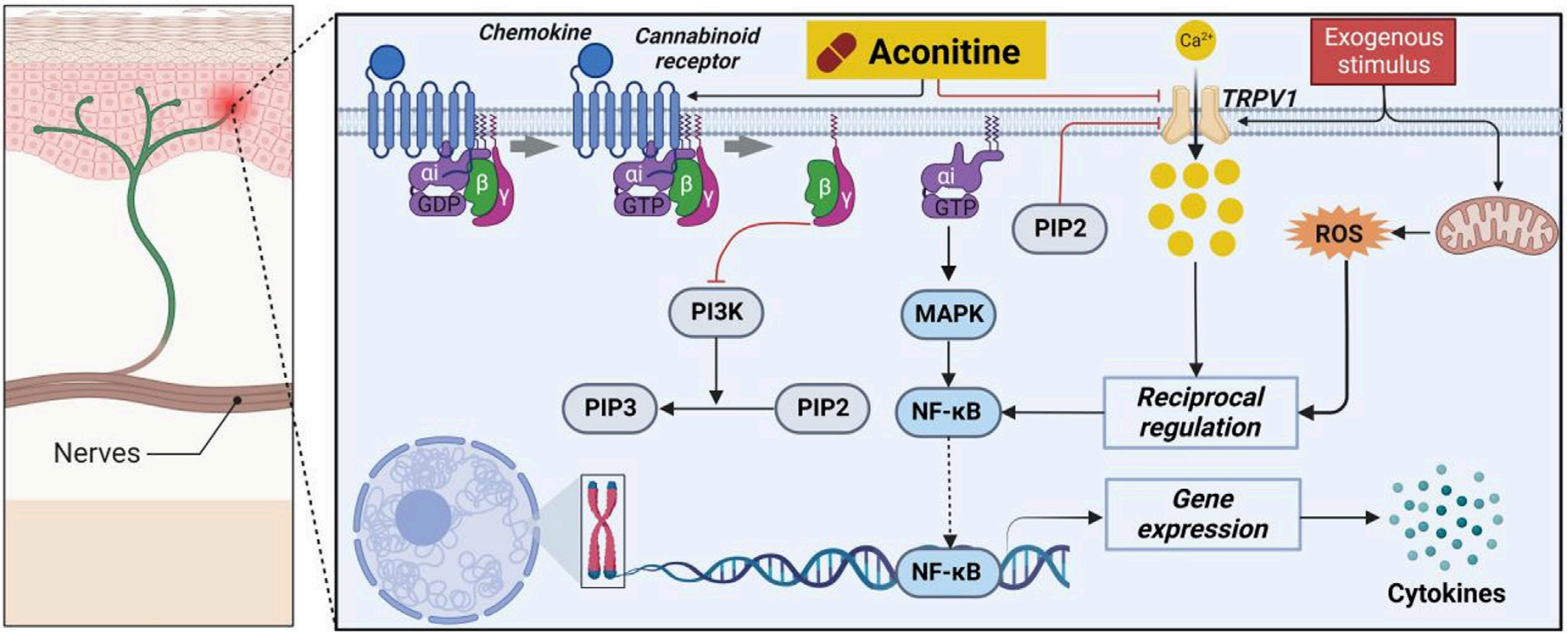
Pharmacological mechanisms of AC-mediated analgesic efficacy. AC possesses an analgesic activity by regulating cannabinoid receptors 1 and 2 (CB1 and CB2) and transient receptor potential cation channel subfamily V member 1 (TRPV1). The black arrow represents the activation of the signalling pathway, and the red horizontal line represents the inhibition of the signalling pathway. MAPK, mitogen-activated protein kinase; NF-κB, nuclear transcription factor-κB; PI3K, phosphatidylinositol-3-hydroxykinase; PIP2, peroxisome proliferation transcriptional regulator; PIP3, 1-phosphatidylinositol 4,5-bisphosphate phosphodiesterase beta-3; ROS, reactive oxygen species.

Numerous scholarly sources have documented the favorable analgesic properties of AC (Luo et al., 2020). Specifically, the antagonistic impact of AC on taste bud tissue biosensors was investigated through *in vitro* studies. As shown in Figure 10, the findings demonstrated that AC, acting as a mixed allosteric regulatory ligand of capsaicin, potentially triggers intracellular G protein/PI3K/PIP2 signaling pathways by binding to CB1 and CB2 (Jin et al., 2023). Consequently, this interaction ultimately leads to an augmentation in intracellular phosphatidylinositol 4,5-bisphosphate (PIP2) levels, consequently inducing the closure of the TRPV1 channel and the manifestation of analgesic effects. Furthermore, the intrathecal administration of AC has the potential to enhance mechanical allodynia and heat hyperalgesia in rats with nerve-ligated neuropathy, with respective ED_50_ values of 35 and 39 p.m. (Zhao et al., 2022). The attenuation of hypersensitivity caused by AC was achieved through the upregulation of spinal microglial dynorphin A expression.

#### 3.4.2 Structure-activity relationship with analgesic efficacy

Among the C_19_ diterpene alkaloids, the majority of compounds exhibiting potent analgesic activity are of the aconitine-type, specifically AC, compounds **15**, **18**, **19**, **37**, **38**, and **39**. Currently, two diterpenoid alkaloids, namely, compounds **15** and **37** have been effectively formulated for clinical application in China (Yin et al., 2016). Moreover, compound **15** exhibited a more potent analgesic effect compared to **37**, as evidenced by empirical findings (Fan et al., 2017). Subsequent investigations into its mechanism of action revealed that the analgesic properties of **15** were associated with alterations in serotonin levels within the brain, as well as the inhibition of ion channels and prostaglandin synthesis (Zhang et al., 2020b). In 2017, researchers conducted a study to examine the analgesic properties and acute toxicity of compounds **15**, **38**, and **39** in mice, utilizing the acetic acid twist method. The findings revealed distinct variations in analgesic effects among the three alkaloids. Specifically, at a dosage of 2 mg/kg, the mice exhibited pain inhibition rates of 81.6%, 58.5%, and 51.2% for compounds **15**, **38**, and **39**, respectively (Liang et al., 2023). Furthermore, the LD_50_ values for the three alkaloids were determined as 4.06 mg/kg, 2.81 mg/kg, and 12.00 mg/kg, respectively.

**FIGURE 11:**
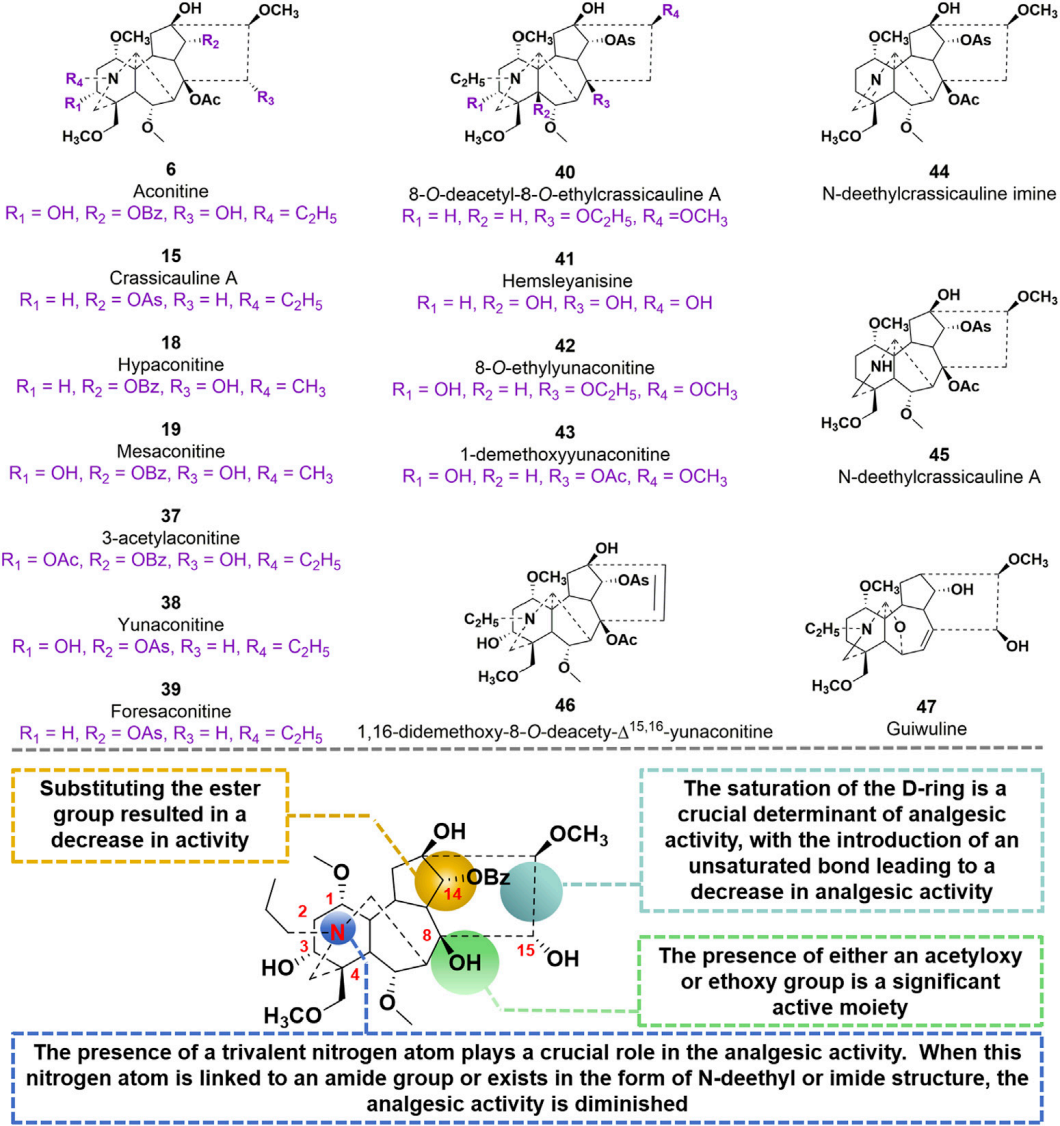
Chemical structures of AC derivatives **37–47** and structural-activity relationship for the analgesic efficacy.

In 2009, compound **15**, as a starting material, was utilized to synthesize 20 derivatives through a semi-synthetic method. The objective of the study was to investigate the analgesic activity of these derivatives and establish a structure-activity relationship (Li et al., 2020). To evaluate the analgesic activity, the researchers employed the acetic acid-induced pain response in mice. The findings revealed that compounds **40–47** exhibited notable analgesic activity, displaying inhibition percentages ranging from 77.8% to 94.1% at doses of 0.1–10 mg/kg after 20 min of administration (Thawabteh et al., 2021). Among these compounds, **40** and **42** demonstrated the most potent analgesic effects, with EC_50_ values of 0.0591 mg/kg and 0.0972 mg/kg, respectively. In comparison, the EC_50_ value of the parent molecule **15** was determined to be 0.0480 mg/kg. Compound **47**, a diterpenoid alkaloid, exhibits notable biological activity. Through a hot plate experiment conducted on mice, it was determined that this compound possesses analgesic properties, with an EC_50_ value of 15 mg/kg. In comparison to AC, which has an EC_50_ value of 0.08 mg/kg, compound **47** demonstrates inferior analgesic efficacy. However, it is noteworthy that compound **47** exhibits significantly lower toxicity, as evidenced by its LD_50_ value of 500 mg/kg, in contrast to AC’s LD_50_ value of 0.16 mg/kg. Based on these studies, the SAR of AC derivatives may be summarized as follows: (i) The presence of a trivalent nitrogen atom in the A-ring structure plays a crucial role in the analgesic activity. However, when this nitrogen atom is linked to an amide group or exists in the form of N-deethyl or imide structure, the analgesic activity is diminished; (ii) The presence of either an acetyloxy or ethoxy group at the C8 position in the B ring structure is a significant active moiety; (iii) Substituting the ester group at C14 resulted in a decrease in activity; (iv) The saturation of the D-ring is a crucial determinant of analgesic activity, with the introduction of an unsaturated bond leading to a decrease in analgesic activity (Figure 11).

**FIGURE 12:**
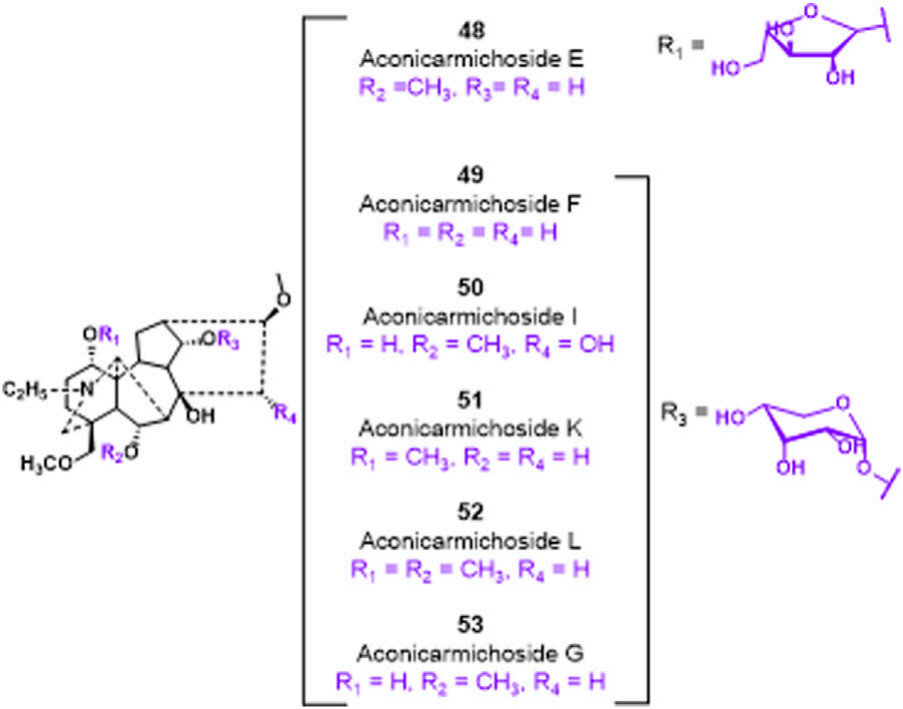
Chemical structures of C_19_-diterpenoid alkaloid arabinosides **48–53**.

Glycosylation, a prevalent and crucial modification of secondary metabolites, significantly contributes to the extensive range of plant natural products. It is worth noting that the glycosylated derivatives frequently demonstrate heightened pharmaceutical efficacy compared to their precursors (Figure 12). In 2018, Guo et al. 2018 identified compounds **48–53**, which possess a sugar unit structure, as potential analgesics. The analgesic properties of compound **48–53** were investigated in acetic acid-induced mice. The findings revealed that compounds **48**, **49**, **51**, **52**, and **53** exhibited higher pain inhibition rates compared to the positive control morphine at a drug concentration of 1.0 mg/kg. The investigation into the structural-activity relationship revealed that the alteration in conformation of the arabinose unit and the presence or absence of a methoxy group at C-6 position did not exert any influence on the analgesic activity. However, the methylation of the hydroxyl group at C-1 position potentially resulted in a reduction in analgesic activity.

### 3.5 Miscellaneous activities

The utilization of aconite and delphine plant extracts as traditional earthwork pesticides has been prevalent for an extended period. These extracts are frequently employed to combat pests and diseases, as well as to eradicate mosquito larvae (Sun et al., 2020). The study observed the insecticidal impact of AC on the rice brown planthoppers (Wei et al., 2019). These studies demonstrated that AC at a concentration of 200 ppm resulted in knockdown rates of 84%, and the LD_50_ for its anti-insecticidal property was determined to be 22.68 ng/pest. Furthermore, the study examined the molecular mechanisms that contribute to the neurotoxic effects of AC on brown planthoppers. Several varieties of diterpenoid alkaloids, such as AC, were obtained through the extraction process from the roots of two aconitum plants. The majority of these diterpenoid alkaloids exhibited significant anti-feeding properties (Shan et al., 2019). The effective concentration at which AC exhibited its antifeedant activity was determined to be 9.2 mg/cm^2^.

## 4 Toxicity

Recently, the increasing usage of Chinese medicine has resulted in incidents of poisoning, raising concerns about the safety of Chinese medicine. As previous mentioned, the toxicity of AC varies slightly when administered through alternative routes, exhibiting an LD_50_ (lethal dose) range of 0.1–0.31 mg/kg. Improper usage of AC can lead to severe poisoning, resulting in various symptoms such as irregular heartbeat, vomiting, queasiness, abnormal heart rhythm, shock, lightheadedness, low blood pressure, and unconsciousness. The primary toxic effects of AC are related to its impact on voltage-sensitive sodium channels found in the cell membranes of excitable tissues like the heart muscle, nerves, and muscles. Additionally, recent studies have also revealed its potential for causing harm to embryonic development, kidney function impairment, liver damage and reproductive health issues. This chapter will explore the diverse toxicological and side effects elicited AC.

### 4.1 Cardiotoxicity

It is believed that the irregular heartbeat caused by AC is mainly due to the disturbance of balance in ions inside the cells, which occurs as a result of the interaction between AC and the ion channels in the heart. Moreover, AC functions as a prevalent activator of voltage-gated sodium ion channels by binding to the α-subunit of the pathway protein. This binding event induces structural modifications in the protein, ultimately leading to the opening of the gate channel (Wang et al., 2020a; Li et al., 2020). Furthermore, it obstructs the sodium and potassium ATPase (NKA), the precise objective of cardiac glycosides, resulting in the generation of acardiotonic or cardiotoxic consequences. As a result, this procedure leads to a prolonged increase in the amount of Na^+^ and a delay in after-depolarization. The Na^+^-Ca^2+^ exchanger is activated in Ca^2+^ influx mode when intracellular Na^+^ levels are elevated, resulting in enhanced transfer of Ca^2+^ from the extraccellular space to the cytoplasm (Sheth et al., 2015). It is important to highlight that the increase in Ca^2+^ levels following Na^+^ overload induced by AC is not facilitated through a slow channel, as this molecule exhibits a notable potency against the L-type Ca^2+^ channel (LTCC). It should be emphasized that the influx of Ca^2+^ after Na^+^ overload caused by AC is not aided by a sluggish channel, since AC demonstrates a distinct inhibitory effectiveness against the LTCC. In cardiomyocytes subjected to AC stimulation, the amplitude of calcium ion oscillation demonstrated a notable decrease. Conversely, the frequency of calcium ion release from the myocardium sarcoplasmic reticulum, triggered by calcium ion concentration, exhibited an increase, ultimately resulting in an elevation of calcium ion concentration within the cytoplasm of cardiomyocytes (Hsu and Liang, 2021). Moreover, the process through which AC enhances the likelihood of induced arrhythmia has been determined as the disruption of AC-induced changes in intercellular Ca^2+^ oscillation patterns that affect the excitation-contraction coupling. The phosphorylated activation of protein kinase Cα (PKCα)-recombinant connexin 43 (Cx43) signal exhibited a negative correlation with the aforementioned interference. It is widely recognized that the excessive accumulation of sodium and calcium ions within cardiomyocytes serves as the primary etiology for arrhythmia induced by AC (Wang et al., 2021b). Multiple recent research studies have suggested a possible link between the arrhythmogenic characteristics of AC and the condition of the potassium channel (Wang et al., 2021b). The human ether-a-go-go related gene (hERG) current, which acts as the main potassium current responsible for repolarization, plays a vital part in regulating the length of action potential in cardiomyocytes (Coulson et al., 2017). When AC is in the open state, it has been discovered to obstruct hERG, but it demonstrates a limited attraction to it in the resting or closed state. Consequently, this leads to a prolongation of myocardial action potential duration (APD) and the induction of early after-depolarization. The Kv1.5 channels are primarily responsible for generating the delayed rectifier potassium current in atrial myocytes. This current plays a crucial role in the outward repolarization during Phase 1 and 2 of the action potential in atrial myocytes (Li et al., 2010; Zhang et al., 2018). AC-mediated atrial arrhythmia can potentially occur due to the blockade of Kv1.5 channels, leading to an elongation of the duration of the action potential and subsequent prolongation of the refractory period in the atrial muscle (Wang et al., 2021c).

**FIGURE 13:**
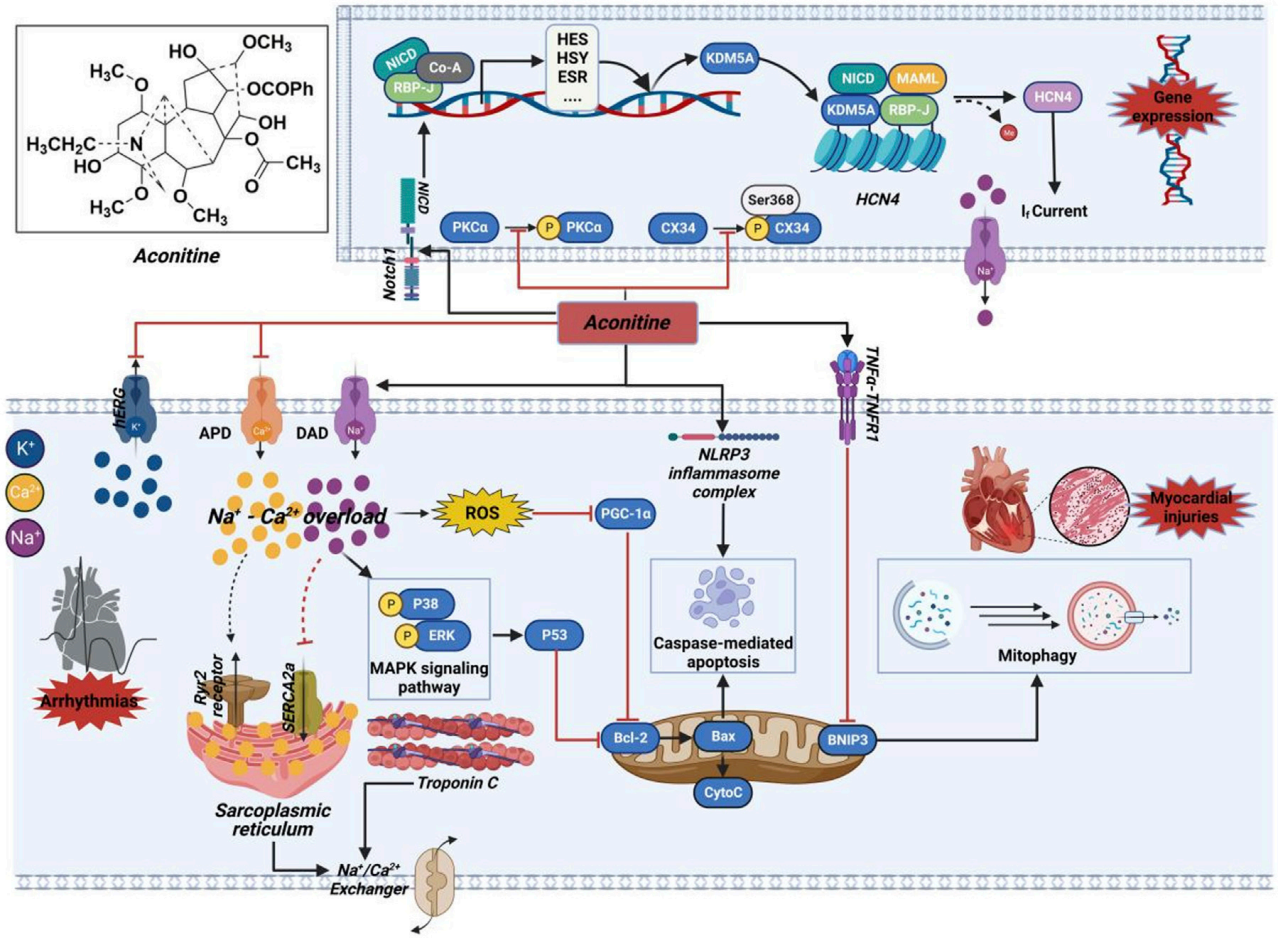
Mechanism of the cardiotoxicity of AC. This molecule potentially exerts its effects through interactions with ion channels, induction of cytotoxic injury, as well as epigenetic and transcriptional regulation. The black arrow represents the activation of the signalling pathway, and the red horizontal line represents the inhibition of the signalling pathway. APD, action potential duration; Bcl-2, B-cell lymphoma-2; BNIP3, BCL2/adenovirus E1B 19 kDa protein-interacting protein 3; Co-A, coenzyme A; CX34, recombinant connexin 43; CytoC, cytochrome c; DAD, delayed after depolarization; ERK, extracellular regulated protein kinase; ESR, estrogen receptor; HCN4, hyperpolarization activated cyclic nucleotide-gated channel 4; hERG, human ether-a-go-go related gene; HES, hairy and enhancer of split; HSY, hydroxyproline-rich systemin; KDM5A, lysine-specific demethylase 5A; MAPK, mitogen-activated protein kinase; MAML, magnetosome protein; NICD, notch intracellular domain; NLRP3, NOD-like receptor thermal protein domain associated protein 3; Notch1, neurogenic locus notch homolog protein 1; PGC-1α, peroxisome proliferator-activated receptor gamma coactivator 1α; PKCα, protein kinase Cα; RBP-J, recombining binding protein suppressor of hairless; ROS, reactive oxygen species; Ryr2, ryanodine receptor; SERCA2a, sarco/endoplasmic reticulum Ca^2+^-ATPase 2a; TNF-α, tumor necrosis factor-α; TNFR1, Tumor necrosis factor receptor 1.

Apoptosis serves as another primary mechanism through which AC triggers cytotoxic damage to the myocardium (D’Arcy, 2019). The excessive buildup of cytoplasmic calcium ions promotes the production of reactive oxygen molecules, leading to lipid peroxidation of the cell membrane and oxidative harm to DNA. Ultimately, these processes culminate in the induction of cardiomyocyte apoptosis (Wang et al., 2021d). Higher amounts of AC have been found to directly increase the levels of mitochondrial superoxide anion while simultaneously reducing mitochondrial membrane potential (Sun et al., 2014). The changes have been associated with the decrease in peroxisome proliferator-activated receptor gamma coactivator 1α (PGC-1α) expression caused by AC, along with the development of mitochondrial malfunction and subsequent cytochrome C release into the cytoplasm. As a result, this series of occurrences initiates the activation of the apoptotic pathway mediated by the mitochondria (Wang et al., 2021e). Moreover, the cardiotoxic effects of AC might be triggered by the induction of apoptosis associated with inflammation, as indicated by the notable increase in crucial proteins within the TNF-α and NOD-like receptor thermal protein domain associated protein 3 (NLRP3) inflammasome signaling pathway (Peng et al., 2020). Besides the excessive accumulation of calcium within cells and apoptosis triggered by mitochondria, Caspase-3, an essential agent of cell death, also plays a role in the cytotoxic harm associated with inflammation. An apparent antagonistic relationship between autophagy and apoptosis is observed. Nonetheless, the mechanism of mitophagy that relies on BNIP3, as illustrated in Figure 13, functions to remove impaired mitochondria and counteracts the activation of NLRP3 and TNF-α-induced cell death (Qiu et al., 2021). The suppression of this mitophagy process has been uncovered by AC. The combined results indicate that AC-induced myocardial injury is caused by mitochondrial apoptosis, pro-inflammatory reactions, and oxidative stress, all of which contribute to its proapoptotic effectiveness.

The analysis of zebrafish heart treated with AC using RNA-sequence has shown that the AC-induced inhibition of LTCC results in a notable increase in the expression of a LTCC protein called CAV1.2 (Rai et al., 2017). Furthermore, AC elicits inhibition that modulates the expression of ryanodine receptor (RyR2) and sarco/endoplasmic reticulum Ca^2+^-ATPase 2a (SERCA2a), resulting in heightened release of calcium ions within the sarcoplasmic reticulum and subsequent alteration of calcium ion concentration in cardiomyocytes. Consequently, the activity induced by AC initiates the intracellular calcium signaling pathway. Moreover, there was an observed rise in the levels of B-cell lymphoma-2 (Bcl-2) and various members belonging to the Caspase family. Consequently, the suppression of the corresponding genes of AC resulted in premature cardiac dysfunctions and embryonic mortality (Kondo et al., 2022). Nevertheless, the exact mechanism by which AC influences the levels of the corresponding genes is still not completely comprehended. According to previous study, the repeated use of AC led to the activation of neurogenic locus notch homolog protein 1 (Notch1) signaling, resulting in the formation of a transcriptional complex consisting of notch intracellular domain (NICD) and lysine-specific demethylase 5A (KDM5A). The involvement of this complex is crucial in the methylation process of the hyperpolarization activated cyclic nucleotide-gated channel 4 (HCN4) promoter, resulting in the stimulation of the HCN4 channel and subsequent amplification of the I_f_ current within cardiomyocytes (Zhou et al., 2020). Consequently, AC-mediated ventricular myocardial dysrhythmia occurred, suggesting that AC treatment may regulate gene expression by initiating epigenetic alterations, thereby unintentionally causing cardiotoxic effects.

### 4.2 Neurotoxicity

The neurotoxicity of AC is manifested through its disruption of ion channels, modulation of neurotransmitter levels, interference with cellular energy metabolism, and induction of neuronal apoptosis (Zhao et al., 2010). Specifically, several studies have indicated that AC has the ability to impede neuromuscular transmission and induce depolarization in skeletal muscles of mice and frogs (Chan et al., 2021). The acute spinal neurotoxicity induced by AC manifests as a spectrum of abnormal motor behaviors, including cyanosis, weakness, abdominal breathing, particularly relaxation paralysis. These adverse effects become apparent within 2–3 min and persist for 5–10 min, during which the mortality rate significantly escalates in affected individuals (Li et al., 2016). Moreover, AC has been demonstrated to induce neurotoxicity by regulating 5-hydroxytryptamine receptors and can also elicit epileptoid activity in the cerebral endocortex of animal models, exhibiting acute and prolonged excitatory effects (Chen et al., 2021). Additionally, a study suggests that AC may inhibit delayed rectification of potassium ion currents in differentiated neuronal cells, potentially leading to abnormal neuronal excitation (Çankal et al., 2021). Notably, AC can disrupt neurotransmission pathways, impeding the passage of neuronal signals through synapses and resulting in impaired limb control or paralysis (Chan et al., 2021). Recent study have demonstrated that AC-induced neurotoxicity is associated with excitotoxic mechanisms mediated by glutamate and aspartate, subsequently leading to lactic acid accumulation and glucose depletion (Zhang et al., 2023).

In 2022, the neurotoxic targets of AC were predicted and analyzed using network pharmacology and molecular docking techniques (An et al., 2022). A variety of molecular biological experiments have demonstrated that AC induces neurotoxicity by modulating multiple pathways, including the MAPK signaling pathway and AKT protein-related pathway, disrupting cell membrane integrity, impairing mitochondrial function, affecting cellular energy metabolism, and inducing apoptosis. Nissl staining and TUNEL experiments further confirmed that AC promotes neuronal apoptosis and reduces neuron count as indicators of its neurotoxic effects. Molecular docking investigations provide valuable insights into the binding conformations of ligands to target molecules by determining the molecular interactions involved. The results revealed strong binding affinity between AC and key target proteins serum albumin (ALB), AKT1, and Caspase-3 with stable conformation at the binding sites, supported by protein binding energies below −7.0 kJ/mol. In 2023, To investigate the mechanisms of interaction between human serum albumin (HSA) and AC derivatives benzoylaconine, a comprehensive approach involving multi-spectroscopic analysis, molecular docking, and dynamics simulation was employed (Zhou et al., 2023). The results obtained from molecular docking revealed that, apart from hydrogen bonding and van der Waals forces, hydrophobic interactions played a crucial role in the formation of the HSA-benzoylaconine complex. Additionally, specific residues, namely, Trp-214, Leu-219, Leu-238, and Ala-291, were found to significantly contribute to the binding of benzoylaconine to HSA. This study aims to clarify the distribution and metabolism of AC, thereby establishing an empirical foundation for investigating the toxicological and adverse effects associated with AC.

### 4.3 Others

Numerous studies have demonstrated that AC exhibits fetal, reproductive, and hepatic effects. Specifically, at a dose of 5 mg/L, significant somatic abnormalities and developmental defects in vital organs were observed in embryos. Therefore, strict dosage control or substitution with a safer treatment regimen is imperative when administering AC to pregnant women. *In vitro* placental models demonstrate that AC exerts reproductive toxicity by modulating amino acid metabolism in the placenta. Further mechanistic investigations reveal that AC downregulates the expression of androgen synthesis-related proteins SCA1, HSD3B1, CYP17A1, and HSD17B3. Moreover, AC induces mesenchymal cells apoptosis through reactive oxygen species generation (Wang et al., 2019). The enhancement of cytotoxicity for specific drugs can be achieved through the induction of autophagy. Experimental evidence has shown that AC effectively induces hepatic autophagy in mice by modulating the phosphorylation levels of upstream proteins, such as PI3K, AKT, mTOR, 70S6K, and 4EBP-1 (Greco et al., 2023).

Fortunately, through the advancements in science and technology, researchers have discovered several approaches that can effectively mitigate the toxic and side effects of aconitine while enhancing its pharmacological efficacy (Hou et al., 2023). These methods encompass traditional Chinese medicine treatments such as decoction and boiling, synergistic combinations with other Chinese medicinal compounds, as well as modifications in dosage forms (Chen et al., 2020). Additionally, further investigation is required to decrease the toxicity of Chinese medicine and validate its rationality and compatibility.

## 5 Conclusion and future perspectives

*Aconitum carmichaelii Debeaux*, commonly known as Fuzi, is a renowned TCM employed for the management of cancer, inflammation, and other ailments. Its primary active constituents are aconitine-type C_19_-diterpenoid alkaloids, which exhibit various pharmacological properties including antitumor, anti-inflammatory, cardiotonic, and analgesic effects. Extensive efforts have been dedicated to the discovery of derivatives that possess high selectivity, remarkable pharmacological activities, and minimal adverse reactions. This review provides a comprehensive summary of the molecular mechanism underlying the biological activities, analogues, and structural-activity relationships of AC. The knowledge presented herein will undoubtedly prove advantageous in the future development of novel drug-like derivatives. However, it is important to note that this review lacks clinical explorations pertaining to the pharmacological activities of AC. Consequently, future research endeavors should prioritize the execution of the following tasks.

1) Target discovery plays a crucial role within the realm of drug development. It serves as the initial step in the target-based drug development process, encompassing the stages of disease identification, target discovery, seedling compound screening, lead compound optimization, animal experiments, preclinical experiments, and clinical experiments. Target discovery serves as the foundation for drug screening and design, yet it is worth noting that numerous drugs with unidentified targets exist, potentially concealed within other drugs or yet to be recognized as viable targets for therapeutic intervention (Hicks et al., 2020). Despite the identification of numerous pharmacological activities associated with aconitine-type C_19_-diterpenoid alkaloids, the precise molecular targets remain elusive. Consequently, there is a need to elucidate the underlying regulatory network involved in these activities. Currently, it is imperative to investigate the interaction and functioning of small drug molecules within biological macromolecules in the human body, a process commonly referred to as “target fishing.” In the realm of biological experimentation, a range of chemical genetic techniques can be employed to identify potential drug targets (Hati et al., 2021). Moreover, computational biology can play a pivotal role in expediting the identification of prospective drug targets, when coupled with experimental screening, thereby expediting or even directing the target discovery process (Qiu et al., 2020).
2) As an exceedingly toxic substance, an overdose of AC would elicit a range of cardiotoxic consequences, such as polymorphous arrhythmias and damage to the myocardium. Interestingly, recent evidence has demonstrated that even minimal quantities of AC are necessary to attain the desired cardiac efficacy of AC-containing medications. Notably, crude aconite (Fuzi) is commonly employed as a cardiotonic agent in urgent medical situations. These discoveries suggest a significant therapeutic potential for AC and/or its metabolites. Nevertheless, further research and the subsequent creation of AC-derived pharmaceuticals are necessary.
3) The development of a drug necessitates the establishment of a suitable balance between its efficacy against disease-related therapeutic targets, physicochemical properties, pharmacokinetic characteristics, and safety, thereby indicating its potential for future advancement (Norman, 2020). Aconitine-type C_19_-diterpenoid alkaloids exhibit intriguing chemical structures and potent pharmacological effects, yet their clinical application has been significantly restricted due to challenges such as poor water solubility, limited oral bioavailability, and an unclear molecular mechanism of action (Ding et al., 2016). Ongoing endeavors are necessary to advance the synthesis of novel analogues in order to enhance the therapeutic efficacy of aconitine-type C_19_-diterpenoid alkaloids and broaden their understanding of structure-activity relationships. In specific instances, alteration of particular substituents within these fundamental frameworks has been observed to modulate disease-related proteins. Specifically, analysis of the pharmacophore of aconitine-type C_19_-diterpenoid alkaloids has highlighted the essential role of the key molecular skeleton in the development of bioactive molecules. The pursuit of small molecular compounds derived from natural products’ structures is a significant approach in the field of drug discovery. A prime illustration of this is the discovery of compound **27**, achieved by simplifying and modifying the aconitine scaffold. Furthermore, the advancement of structural biology and computational chemistry has ushered in a new era in the investigation of medicinal plants and natural sources. Notably, computation-based methodologies offer a promising avenue for the identification of natural product-based medicines.
4) Previous clinical studies have been conducted in China on aconitine-type C_19_-diterpenoid alkaloids. However, further investigation is required to fully understand their clinical efficacy. As a result, the inclusion of clinical experiments is essential for the precise evaluation of their beneficial effects on various therapeutic interventions for diseases. However, the implementation of rigorous triple-blinded randomized control trials is imperative to authenticate the clinical observations. Furthermore, it is of utmost importance to establish a scientific and systematic framework that elucidates the underlying therapeutic mechanisms.
